# A day in the life: Using contextual interviews to understand the health of home-based Mapuche weavers

**DOI:** 10.1371/journal.pgph.0000353

**Published:** 2022-05-10

**Authors:** Gabriela Gracia, Alison Guzman, Linda Forst

**Affiliations:** 1 University of Illinois at Chicago, School of Public Health, Division of Environmental and Occupational Health Sciences, Chicago, IL, United States of America; 2 Research Consultant, Boston University, Boston, MA, United States of America; 3 MAPLE Microdevelopment Chile, Villarica, Araucanía IX Region, Chile; Brigham and Women’s Hospital, GUATEMALA

## Abstract

The craft sector employs 305 million people worldwide, primarily women, rural and home-based workers. Home-based weavers, an understudied workforce, are subject to a range of hazards and adverse health effects related to their weaving work and domestic responsibilities (e.g., housework, farm work, childcare, eldercare). The Mapuche represent Chile’s largest Indigenous group with about 20 percent residing in the Araucanía region, where agriculture and tourism, including craftwork, are important sources of income. Using a purposive sampling approach, we conducted Spanish-language contextual interviews (N = 10) with Mapuche weavers across four communities, allowing us to observe participants in their home settings, watching them weave and discussing tasks, decision-making, and behaviors during the work process. Participants ranged in age from 29–55 years. A combination of semi-structured, audio-recorded interviews, videos, photographs and written observations yielded a time-wheel of a typical day for each weaver; the types of weaving and non-weaving work (including hours per day); workspace visuals; self-reported health problems, causes and treatments; exposure hazards; and potential ergonomic interventions. In addition to weaving, housework (n = 10), farm work (n = 7), wool production (n = 7), natural and chemical dyeing (n = 7) and child-rearing (n = 4) were identified as work activities. The most commonly cited weaving-related health problems were eyestrain (n = 7) and pain in the back (n = 6), shoulder (n = 5), arm (n = 4), hand (n = 4), neck (n = 3), wrist (n = 3) and fingers (n = 3). When asked to identify potential improvements to their weaving workspace, participants identified the need to having a dedicated workspace for weaving (n = 7), improving their existing workspace with better seating (n = 4), lighting (n = 3), insulation (n = 2) and increasing the size of their workspace (n = 2). This methodology, blending traditional occupational health tools with qualitative methods, was instrumental in understanding the range of hazards associated with home-based work and identifying potential ergonomic interventions for this global workforce.

## Background

### The Mapuche people

The Mapuche people represent Chile’s largest Indigenous population, estimated at about 1.7 million or roughly 80% of the Indigenous population and 10% of the total population in Chile [1, 2]. The Mapuche endured significant struggles in Chile dating back to the Pacification of the Araucanía (1861–1883). This territorial campaign by the Chilean government forced the Mapuche into reservations while their land was sold to Chilean and foreign settlers for agricultural development [3, 4]. The loss of land and livestock radically altered the Mapuche way of life; instead of being able to live off their land exclusively, many were forced to work as temporary or seasonal laborers [5]. Tremendous disproportionate socioeconomic hardships and systemic discrimination have resulted in long-term effects on the health, wealth, education and representation among the Mapuche [6–10]. And yet, the Mapuche have demonstrated a resolve to maintain their cultural identity and their craft. Their political mobilization have fueled important discussions around Indigenous rights that are currently on display in Chile as a new Constitution is being drafted [11–13]. Today, about 20% of the Mapuche population reside in the Araucanía region and many families continue to live on small, eroded plots of land used for subsistence farming throughout the year [14–16]. The rural area of the Araucanía region, where many Mapuche reside, is among the poorest in the country [6]. The main employment sectors are agriculture, temporary seasonal work, forestry and tourism. Craftwork, part of the tourism sector, represents an important and complementary source of income for Mapuche women, allowing many to work at home and carry out their domestic responsibilities [15, 17–20].

Rural Mapuche women in Chile experience cultural, geographic and language barriers that result in lower educational attainment and higher illiteracy rates compared to their counterparts [21, 22]. Poverty levels are even more pronounced with 28.4% of Indigenous respondents captured in the multidimensional poverty index versus 18.2% of non-Indigenous respondents [23]. Based on this information, it is reasonable to assume that Mapuche women would likely enter into labor markets with a disadvantage, falling into cycles of poverty more so than the general population. Home-based work and the craft sector provide important opportunities for women to gain independence and economic stability.

Home-based workers (HBWs) account for a large portion of the informal economy, with women making up more than half (57%) of the 260 million global home-based workforce [24–26]. The International Labour Organization (ILO) estimates that 29% of workers in Chile belong to the informal workforce [27]. In the Americas, women represent 54% of all HBWs and 71% of women living in rural areas [26]. HBWs typically work by themselves or in small groups with family members helping with work and domestic-related tasks, as needed [28–30].

### The craft sector and Mapuche textiles

An estimated 305 million people work in the craft sector, comprised largely of women, rural and home-based workers [31, 32]. With an estimated market value of $526.5 billion, the craft sector is one of the largest sectors and contributors to the global economy, as well an important avenue for cultural preservation [31–33]. However, difficulties capturing informal and home-based workers, lack of investment and market access are major challenges for this sector [31, 32]. Additionally, artisans cite limited access to raw materials and inadequate workspaces as some of the greatest barriers to economic viability [34]. Women make up a large portion of the craft sector in Chile, with estimates ranging from 64% to 84% [31, 34]. Roughly 38% of Chilean artisans work in textiles and nearly 16% of the craft sector is made up of Mapuche artisans [34].

**Fig 1:**
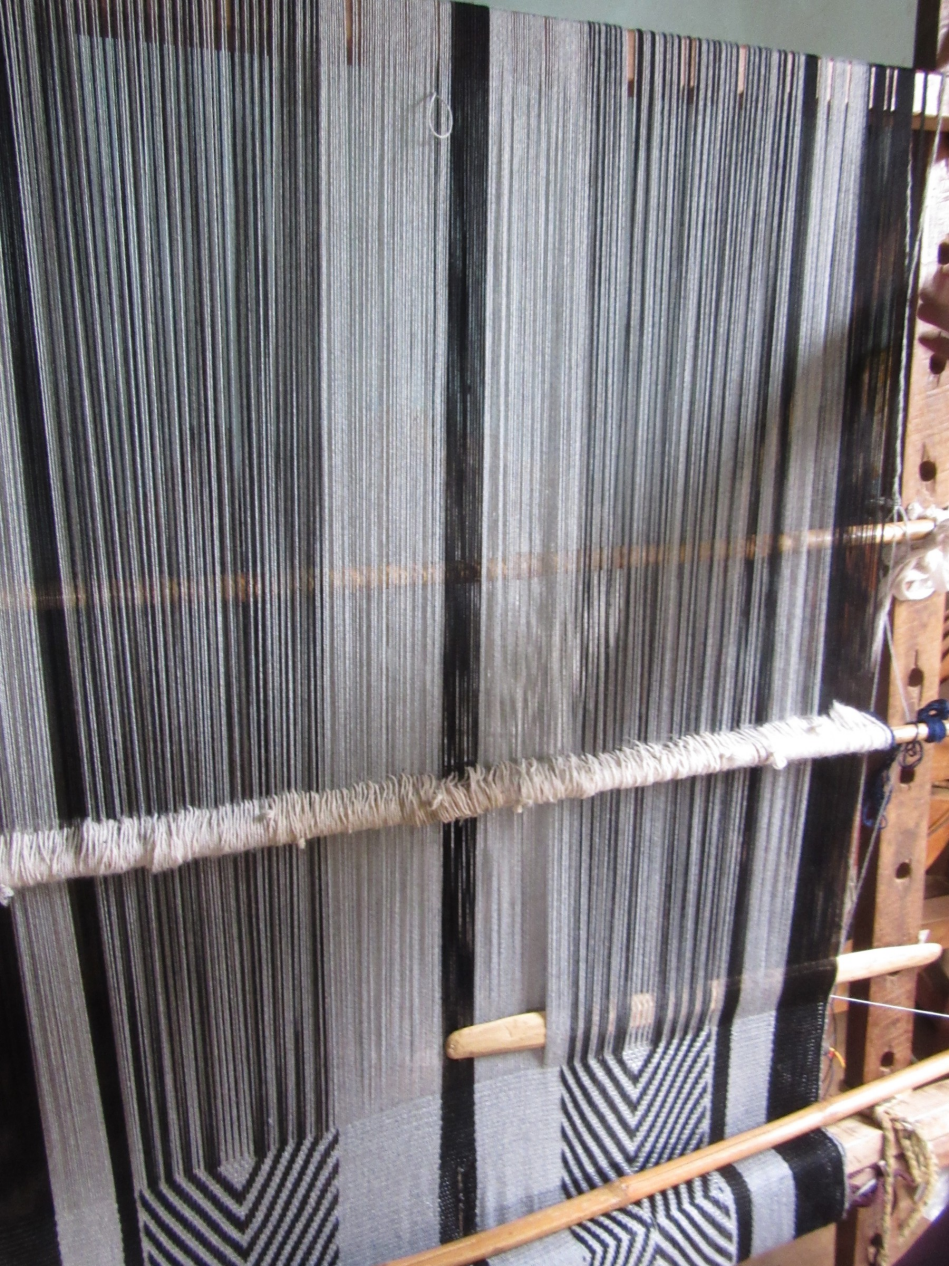
Photograph of an assembled vertical loom (fieldwork photo 2016).

The cultural importance of Mapuche weaving cannot be overstated. As is common for Indigenous weavers in Latin America, women learn to weave from their mothers or maternal figures, and typically weave at home in common areas [30, 35]. Textile work entails wool production, wool acquisition, natural and chemical dyeing, the use of traditional standing looms and Mapuche designs [36–38]. These designs include symbols reflective such as the *cruz andina* (Andean cross), the *anümka* plant and the *rayen* flower [16, 35, 37, 39]. The colors used are inspired by nature, with many women actively involved in wool production and natural dyeing in their homes [35–37]. While Mapuche weavers use both horizontal and vertical looms, this study sample used vertical looms exclusively. Vertical looms are made up of wooden boards (*kilwos)* with ties (*tientos)*, a heddle holder (*tonón)* and a wooden tool called a batten (*ñireo)*. **Fig 1** is a photograph of an assembled loom.

### Weaving and associated hazards

The United Nations Sustainable Development Goal (SDG) 8 emphasizes *“*productive employment and decent work for all,” however, what this means for the craft sector is often times missing from discussions [40]. Weavers experience a wide range of work-related hazards and health issues that have long-term effects on their quality of life. Occupational hygiene characterizes hazards under the following categories: biomechanical, psychosocial, physical, biological and chemical [41]. Biomechanical hazards include repetitive or awkward postures that can lead to musculoskeletal disorders. Psychosocial hazards are characterized by workers having high demand and low control over their work, which creates stressful working conditions [42]. Physical hazards include environmental factors such as excessive noise and extreme temperatures. Biological hazards can be transmitted via air, water or direct contact such as mold and viruses. Exposure to chemical hazards can result in skin irritation or respiratory issues [41]. Awkward postures, repetitive movements, contact stress and poor lighting are among the most common hazards associated with weaving globally [25, 28, 43–45]. Awkward postures include sitting, standing and bending and twisting for long periods and weaving at high and low positions.

Several studies have described how static postures can lead to strain in the back, forearm and legs and knee pain [28, 43, 46–48]. Additionally, weaving at high positions (above the elbow) can lead to prolonged shoulder and upper arm flexion, while weaving at low positions (at or below the elbow) can lead to neck and back strain [49]. Repetitive movements occur while setting up the warp, passing yarn across the warp to create the weft, making designs and finishing the product. These activities can strain tendons and cause soft tissue tears [43, 50]. Banerjee and Gangopadhyay (2003) found a significant relationship between pain intensity and repetitive work (p<0.001) as well as pain intensity and working years (p<0.01) among 50 male handloom weavers in West Bengal [50]. Moreover, in a study of 300 traditional weavers in Northeastern India, Devi (2012) found a relationship between health hazards, home-based weaving workspaces and housework [51]. To inform potential interventions that are ergonomically sound, psychosocially acceptable, and feasible to implement, the goal of this research was to characterize hazards, risks, and adverse health outcomes related to weaving among Mapuche women.

## Methods

### Overview of Non-Governmental Organization (NGO)

The study team partnered with an NGO that worked directly with weavers across four different communities. The investigators met with the NGO one year before the study began to discuss shared concerns around the lack of applied research on artisan occupational health and intervention implementation. Upon invitation by the NGO, the investigators met with the weavers at the NGO headquarters and introduced the study, so that all questions or concerns were addressed. Support from the NGO staff, including the Quality Control Director, herself a Mapuche woman, facilitated conversations and transparency around the goals of the study and the weavers’ participation. The Mapudungun language is associated with South American Indigenous groups and is considered endangered [52]. Based on discussions with the weavers, the decision was made to conduct the interviews in Spanish instead of Mapudungun as many Mapuche people prefer to speak Spanish with each other in more formal gatherings.

The NGO’s mission is to improve economic conditions and preserve Mapuche cultural values by producing high quality textiles conveying Mapuche weaving techniques and designs and offering workers competitive wages. Several weavers are on the NGO Board and one weaver serves as the Quality Control Director. In order for a weaver to work with the NGO, they must be recommended by a current weaver and complete a sample piece using a specification sheet that is reviewed by the Quality Control Director. Weavers have the flexibility to choose the amount of work they want to complete each month. They are paid at the end of the month, based on the products they complete and whether or not they pass the quality control reviews. If there are any problems with the product, the weaver can choose to either re-work the piece without compensation for the extra time involved and return it to the office or leave the product as is and have the price of the wool deducted from their monthly payment. All of the participants in this study worked directly with the NGO.

### Study fieldwork

We previously surveyed 33 weavers who worked directly with the NGO as part of the larger study [53]. The investigators visited participants’ homes to observe and better understand their work and domestic responsibilities. Most were married and relied on their husbands to find temporary, seasonal work in nearby towns doing construction or other hard labor. In addition to working for the NGO, some women dedicated themselves to tourism, traditional arts and crafts, or seasonal domestic work as a complementary source of income [39]. A typical day for participants began at sunrise tending to wood burning stoves used for heat and cooking [16]. The use of wood burning stoves coupled with poor ventilation results in heavy smoke inhalation throughout the day. The effects of wood burning stoves on respiratory health is outside the scope of this paper, but has been the subject of intervention research [54, 55].

Most women had farm animals and gardens that they tended to in the morning and throughout the day and organized their days around caring for immediate and extended family members, cooking, cleaning, laundry (done by hand) and weaving. Depending on their schedule, weaving work occurred in the morning and early afternoon, when the children were at school, or in the late hours of the night. Only a few women had a separate workspace, allowing them to weave without interruptions, while most had to work in common areas (living room, dining room, kitchen).

### Study population

From the original sample of 33 weavers, we applied purposive sampling to identify 10 of those weavers who lived across four communities and were representative in terms of weaving experience (2–10 years, 11–25 years, 30 years or greater), weaving products (shawls, mantas, dresses, scarves), weaving techniques (with and without design), workspace types used (living room, dining room, kitchen, separate dedicated workspace), amount of time working with the NGO (less than 1 year to 1 year, 2–5 years, greater than 5 years) and health complaints (eyestrain or eyesight problems, headaches, back pain, shoulder pain, neck pain, wrist pain, finger pain). The study was described to the weavers and written informed consent was obtained by the principal investigator prior to the interviews. Approval was granted by the University of Illinois at Chicago (UIC) Institutional Review Board (IRB) (Protocol #2015–0833) and the Universidad Católica de Temuco IRB.

### Contextual interviews

Contextual interviews allow the researcher to observe participants while working, as well as interact and ask clarifying questions around tasks, decisions and behaviors while they are occurring [56]. For participants, it can be easier to explain and demonstrate tasks while they are working. This structure may also prompt participants to discuss tasks or actions that they may not think are important or forget to describe during a traditional interview [56]. The roles of the researcher and participant are such that the participant is viewed as the “expert” while the researcher is the “student” or “apprentice” who is trying to better understand, learn or do the work process. This dynamic helps facilitate a dialogue around identifying improvements or interventions to address any challenges the participant may be facing [56, 57].

### Survey instrument and data collection

The investigators (native Spanish speakers) developed a Spanish-language, semi-structured interview guide, based on prior knowledge, that focused on workday organization; rationale for workday schedule; weaving skills, experience and workspace; and self-identified health problems, and perceived causes and potential solutions to hazard prevention. In a pilot test, the interview guide was administered to five weavers at the company’s office to check for understanding and clarity of questions and was adjusted, as needed. Each of the 10 audio-recorded sessions lasted three to six hours.

As part of the interviews, the investigators observed participants weaving in their respective workspaces and asked them about daily activities; any discomfort or pain associated with these activities; weaving workspaces; and wool production and dyeing processes (natural and chemical). During the observations, participants were asked about their workspaces, as well as the pieces they were currently working on (e.g. steps to make the piece, length of time to typically complete the piece, etc.). Additionally, the investigators made written observations and obtained video and photographs throughout the interviews. The English and Spanish-language interview guides are provided in the S1 File and S2 File respectively.

For each interview, one investigator developed a ‘Day in the life’ time-wheel visual to capture start-of-day through end-of-day activities, including weaving and non-weaving activities and seasonal variations, with estimated time for each [58]. From there, the investigators asked, “Tell me what you do on a typical day” and then filled in the interviewee’s responses. Next, the time-wheel was reviewed together and any gaps in time were addressed. Once the time-wheel was completed, the investigators asked about seasonal changes in work (weaving and non-weaving) that were also captured in the time-wheel. In addition to documenting daily activities, the graphic model helped facilitate discussions with the weavers around time management, health issues, potential causes and treatments or interventions. Data from the time-wheel were summarized to obtain total hours and averages associated with each task.

### Data analysis

Data were analyzed qualitatively in Spanish. One investigator developed a preliminary codebook that included code definitions, inclusion and exclusion criteria and examples (S3 File). A second investigator reviewed the codebook and the codebook was revised to reflect feedback. The audio-files were uploaded into Atlas.ti (version 7) and one investigator transcribed the interviews verbatim in Spanish. The two investigators coded all ten interview transcripts independently, met to review coding disagreements and came to a consensus. All body diagrams were developed and tested with participants as part of the initial phase of the study (n = 33). Hand diagrams have been previously tested in ergonomics research [59].

## Results

Ten weavers between the ages of 29 and 55 years were interviewed. **Table 1** presents an overview of demographic characteristics, by weaver.

**Table 1:** Demographic characteristics of participants.

Weaver	Age	Marital Status	No. of Dependents	Time with NGO	Years of Weaving Experience	Workspace Type
1	37	Married	3	<1 year	14	Living/Dining Room and Outside Kitchen
2	46	Single	0	>5 years	35	Living/Dining Room and Storage Shed
3	55	Married	1	>5 years	12	Dining Room
4	51	Married	0	>5 years	39	Dedicated Workspace and Kitchen
5	53	Married	2	2 years	10	Kitchen
6	29	Couple (not married)	2	5 years	8	Dedicated Workspace
7	40	Married	2	2 years	2	Kitchen and Living Room
8	47	Married	0	1 year	30	Living Room
9	39	Couple, not married	2	2 years	25	Living/Dining Room
10	51	Married	0	1 year	44	Dining Room

**Fig 2:**
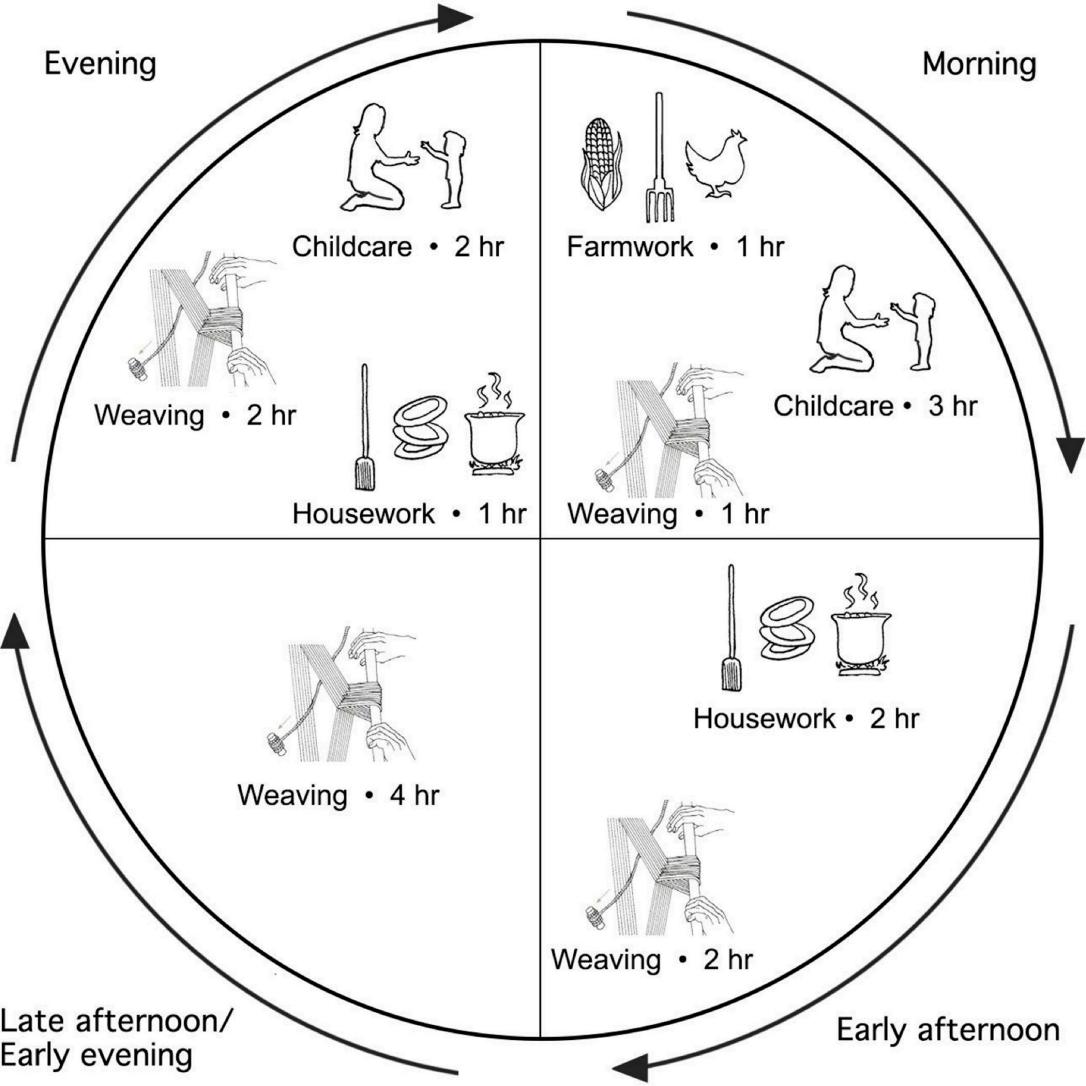
Sample time-wheel visual.

**Fig 2** is a sample graphic of the time-wheel visual. This visual depiction of the workday facilitated discussion related to the issue of multi-tasking and goal-setting. All respondents discussed multi-tasking while weaving—with housework, farm work and caring for children as the most common partnering tasks. For instance, one weaver described a typical day as follows:

*“Then I make lunch and while lunch is getting ready, I go back to weaving. I weave over there and then I get up, check on the food and then peel the potatoes, if I need to.”*

### Work activities: Weaving, non-weaving and seasonality

Activities depicted in the time-wheel were further broken down into weaving versus non-weaving work and seasonal variations. Interviews were conducted in the fall and therefore farm work activities entailed caring for livestock. A small subset of participants (n = 3) had spouses who worked seasonal jobs in the north, requiring them to be gone for extended periods of time, picking fruit in the summer and harvesting potatoes in the fall. When work had to be completed for a deadline, the weaver would indicate the start and end times of her day.

### Non-weaving work

**Fig 3:**
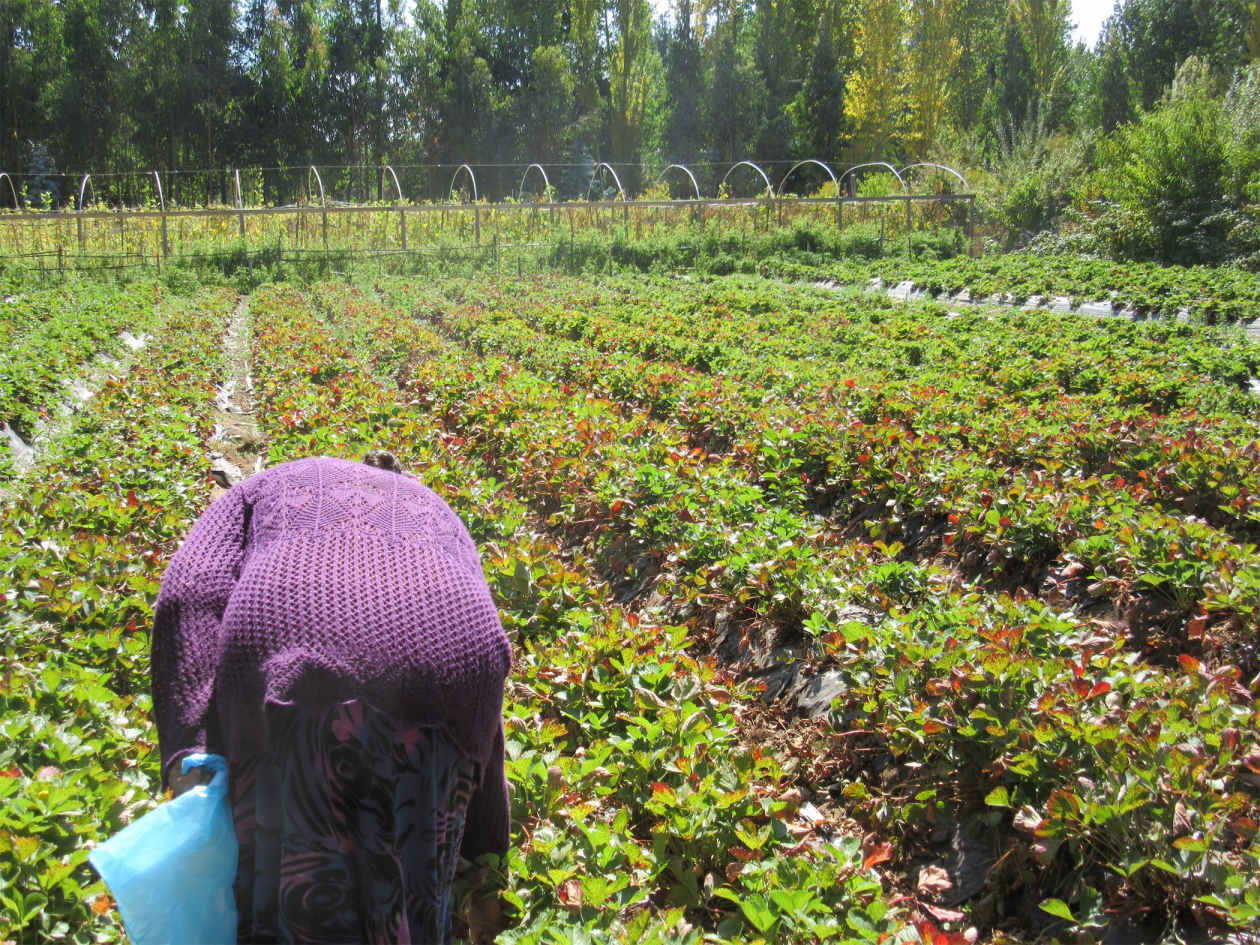
Photograph of a weaver working on her farm (fieldwork photo 2016).

Interviewees identified housework (n = 10), farm work (n = 7) and childcare (n = 4) as the most common types of non-weaving work. The housework activities included cooking, cleaning, doing laundry by hand, and collecting firewood. In a typical day, the estimated time devoted to housework ranged from 1.3–4.5 hours with an average of 3 hours per day. Most respondents (n = 9) own farmland used for family subsistence. Throughout the year, respondents care for livestock, including raising cows, chickens, sheep, pigs, turkeys and horses. However, during the spring and summer months, they also grow fruits and vegetables such as strawberries, potatoes, lettuce, tomatoes, beans, chard, carrots, cucumber, cilantro, and parsley. During the fall months, the estimated time devoted to farm work ranged from 0 to 6 hours with an average of 1.8 hours/day. Six of the nine respondents discussed seasonal changes associated with farm work including sowing (n = 2); watering in the mornings and evenings (n = 3); and harvesting at the end of the summer season (n = 2). **Fig 3** depicts a participant working on her farm. A smaller sample of respondents (n = 4) identified caring for children or grandchildren as part of their daily activities. Among these respondents, time devoted to childcare ranged from 1.5–4.5 hours/day with an average of 3.1 hours/day.

### Weaving and weaving-related work

In addition to weaving for the NGO (n = 10), half of respondents (n = 5) worked for other weaving organizations and cooperatives or sold their products directly to consumers. Additionally, most weavers participated in wool production (n = 7) and natural and chemical dyeing (n = 7) activities. Finally, three weavers worked as weaving instructors and two of the weavers worked for the NGO in other capacities, with one weaver working as the Quality Control Director and one weaver sewing tags on products. Time devoted to weaving ranged from 4–13.5 hours/day with an average of 7.2 hours/day. Wool production and dyeing capture all the activities that occur before weaving can begin. Typically, male household members shear the sheep, or help carry the wet wool during the dyeing process, while the women clean, wash, dry, spin and twist the wool. Among respondents who participate in wool production (n = 7), five discussed summer-specific activities including sheep shearing and washing the wool (n = 4), spinning wool (n = 1), and natural dyeing (n = 2).

### Weaving schedules and motivation

Discussions around the time-wheel also focused on participants’ weaving schedules, with four participants explaining that they had to weave in the evenings due to a heavy weaving workload (more common when there was a deadline) and four other participants stating that they were not able to work in the evenings due to poor workspace lighting and eyestrain. Other weavers worked throughout the day. If the work wasn’t completed by nightfall, they would work through the night.

Participants described the advantages of working at night, with one participant describing:

*“It’s like you’re closed off, you’re not worried about the chickens or sheep, making lunch, nothing. I turn the radio on and I focus on my weaving. At night, you’re more relaxed and you work faster, but time moves more slowly and you’re more productive. That’s why I like to weave at night.”*

Interviewees were asked, “What motivates you to weave?” or “Why do you weave?” All respondents identified economic or financial opportunities as their main motivation, followed by enjoying weaving (n = 7), independence (n = 5) and the ability to work at home (n = 3). Specifically, respondents described contributing financially to the household (n = 7), paying for their children’s education (n = 4), and the independence to make purchasing decisions (n = 2) as economic motivators. Financial independence and educating children were important themes, with one participant asserting:

“*I can help pay so it’s a joy and it’s like someone who didn’t have anything*, *she leaps and suddenly*, *she finds a job and she values herself*.*”*

And another participant sharing:

*“It also helps [to pay for] education, to give my daughter an education…I always tell my daughters that I couldn’t study, but I want to see myself reflected in them one day. When they finish their middle school it’s like I will be finishing it (laughing) even though I know it’s for them”*

### Weaving workspaces

**Fig 4:**
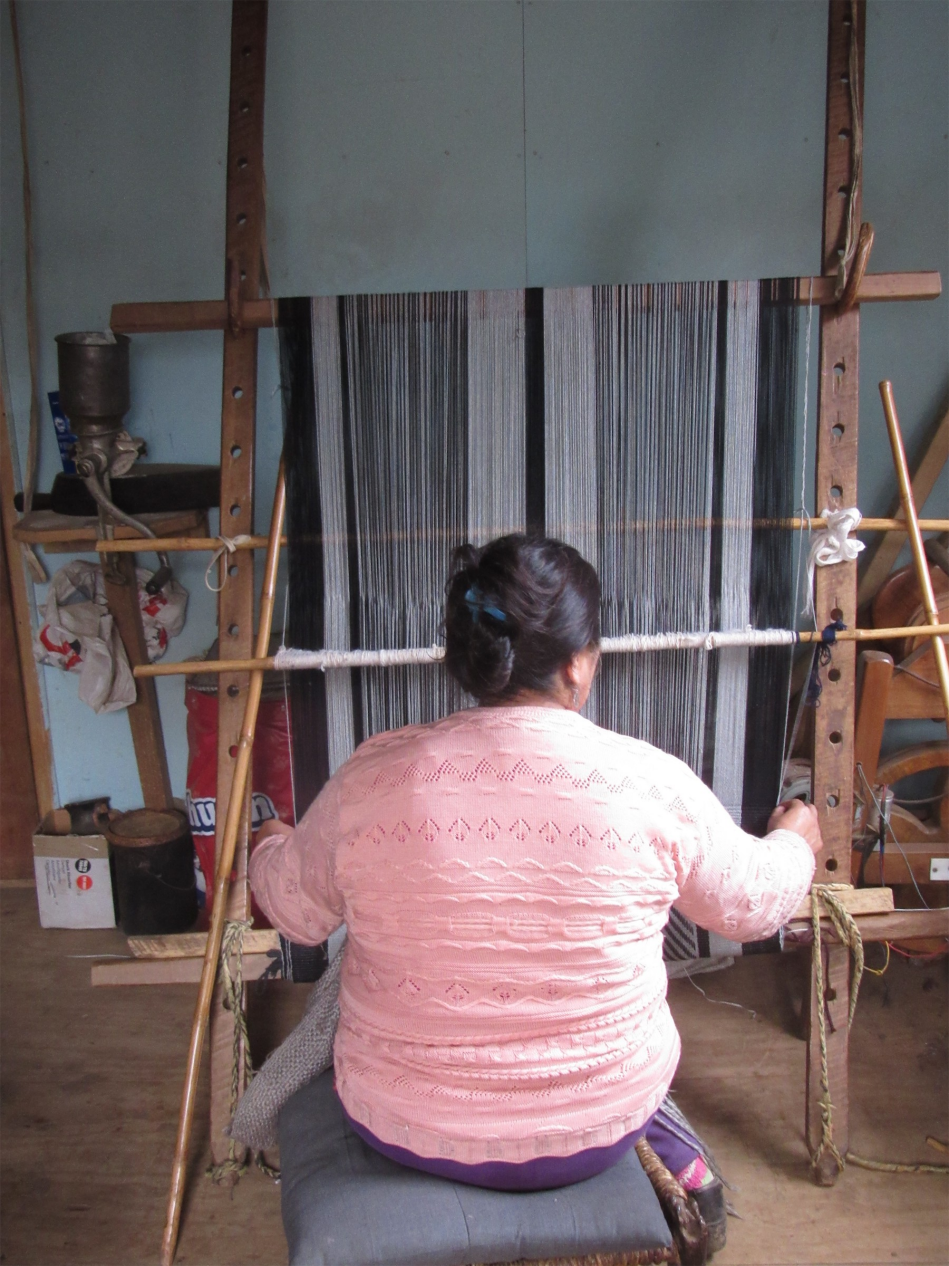
Photograph of a weaver’s workspace (fieldwork photo 2016).

Half of participants used one space exclusively while the other half used at least two workspaces to accommodate weaving large pieces (n = 2) and working more comfortably in heated rooms (n = 2). **Fig 4** is a photograph of a weaver using a dedicated work space next to her kitchen. Seven weavers used the living or dining room area as a common area workspace. Given the shared nature, most workspaces were positioned up against a wall with a vertical loom and a small stool. Weavers described their workspaces as cramped and shared the inconveniences of assembling and dismantling the loom daily, having to remove the loom and textiles during food preparation and cleaning and not having enough room to create the warp. The investigators noted that workspaces measured between 15–25 m^2^. One weaver shared that she had to create the warp in an outside storage unit used for farming equipment because she did not have sufficient space inside her house. Two of the weavers had dedicated weaving workspaces that were located directly outside their homes. They described the benefits as having more space to work, being able to leave their loom and work intact, having less distractions and the general appeal of having a space where they could focus on their work. However, both participants described their workspaces as cold, lacking insulation, with one workspace observed as having an exposed roof. During the fall and winter months, both women described working in very cold, uncomfortable conditions or having to work inside their homes for heat. Discussions around the workspace facilitated discussions around the hazards, adverse health effects, potential causes associated with the workspace and weaving work and possible solutions. This information was used to identify potential interventions as part of this project.

### Suggested ergonomic interventions

**Fig 5:**
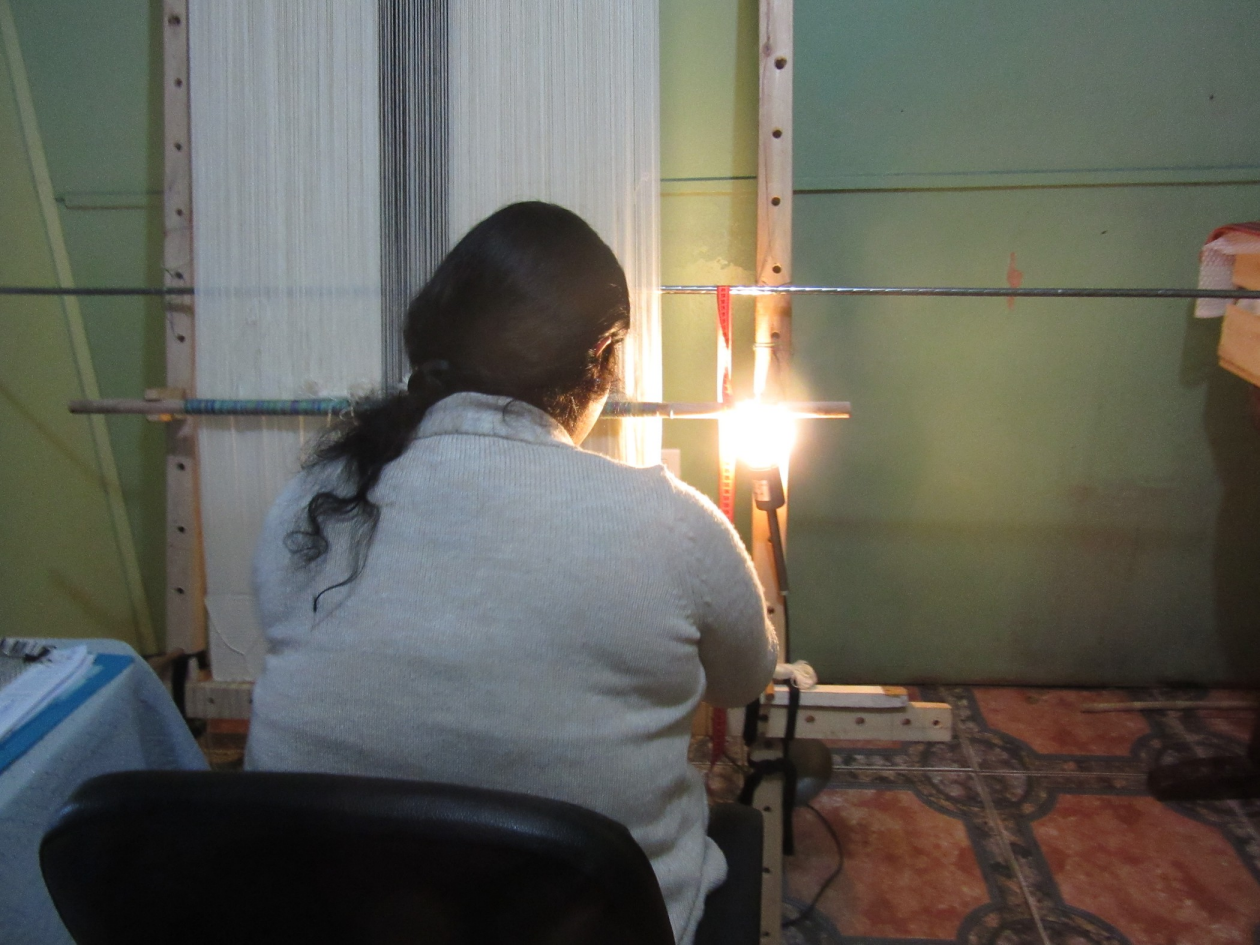
Photograph of a weaver’s loom and makeshift lighting attachment (fieldwork photo 2016).

During the interviews, participants were asked, “What would make your weaving work more comfortable?” and “Are there any changes you would make to your workspace?” Most participants responded by having a dedicated workspace for weaving (n = 7); followed by improving their existing workspace with better seating (n = 4), lighting (n = 3), insulation (n = 2) and enhancing the size of their workspace (n = 2). The non-ergonomic interventions identified by participants included: changes to their working relationship with the NGO, including better pay (n = 3), more relaxed quality control measures (n = 2), not splitting wool (the need to manually divide wool into individual plies to weave fine products) (n = 2), having a work contract (n = 1) and covering transportation costs to and from the office (n = 1). **Fig 5** is a photograph of a weaver working with a light attached to her loom.

This information was summarized and communicated to the weavers and a Participatory Ergonomics (PE) team in order to identify and implement ergonomic solutions. Potentially effective interventions were: better ergonomic conditions, including enhanced lighting; a careful work-rest cycle; changing work postures; lightening heavy workloads by getting the yarn supplied externally and using wool that is stronger and less breakable; finding an insulated workspace outside the home; and getting paid by the hour rather than by the piece. Based on the findings of this study and a review by the PE team, an intervention trial was conducted in this workforce. As part of the study intervention, the PE team carried out an iterative process involving reviewing the project data to prioritize health problems (triangulated with these artisans and the 23 artisans from the original sample), identifying and presenting potential interventions to study participants for feedback and to ensure they were culturally appropriate [53]. These interventions will be described in a later publication.

### Perceptions of health and illness

**Fig 6:**
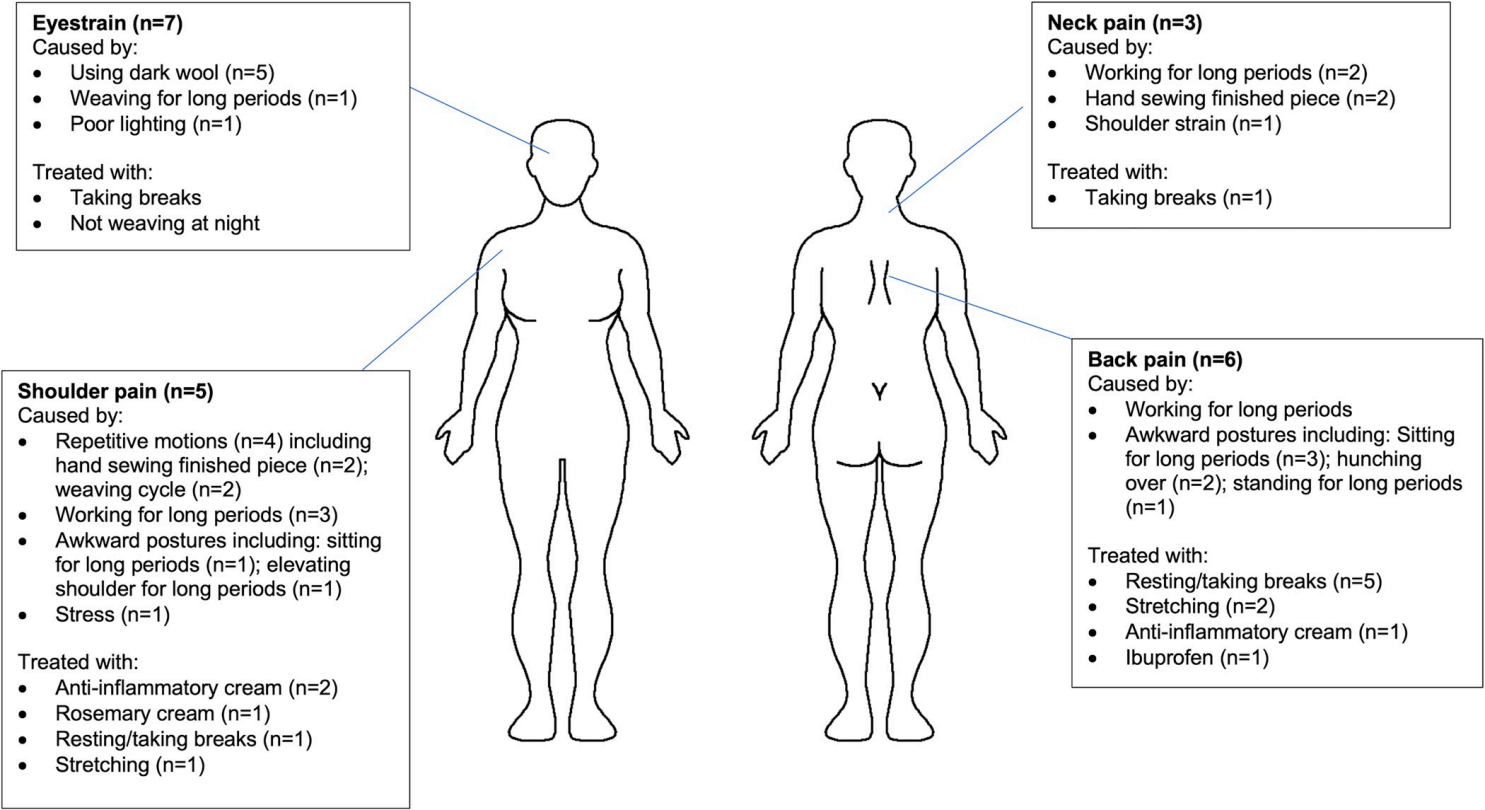
Weaving work: Self-identified health problems, causes and treatments for eyestrain, shoulder, neck and back pain.

**Fig 7:**
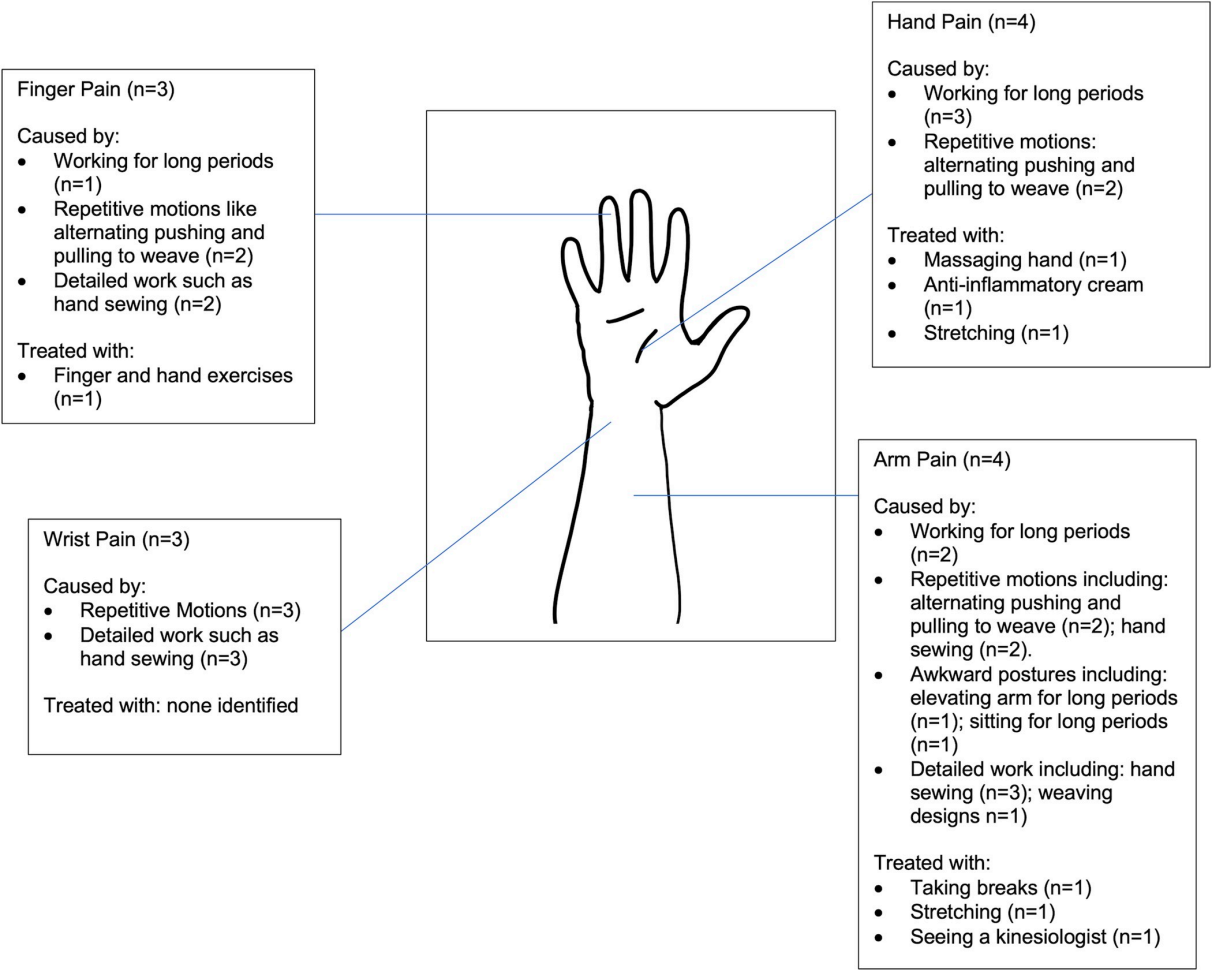
Weaving work: Self-identified health issues, causes and treatments for finger, wrist, hand and arm pain.

Throughout the interviews, participants were asked about health problems associated with their work (weaving and non-weaving) as well as potential causes and treatments. The most commonly cited health issues were eyestrain (n = 7), back pain (n = 6), shoulder pain (n = 5), arm pain (n = 4), hand (n = 4), neck pain (n = 3), wrist pain (n = 3) and finger pain (n = 3). When asked about potential causes, the most common response was weaving-related activities (Figs 6 and 7). When describing the relationship between weaving designs and hand pain, a participant shared:

*“Yes, the design because you’re constantly lifting strands of wool. Every time you have to pass the wool through you have to lift the wool strands and it’s like the most difficult thing to constantly be lifting strands”*

### Hazards

These interviews also provided an opportunity for the investigators to observe and document weaving- and non-weaving-related biomechanical, physical, psychosocial, chemical, and biological hazards. Table 2 is a summary of the most common hazards identified by participants. In terms of weaving-related activities, the most commonly cited activities were weaving for extended periods of time without breaks (n = 8), weaving at night (n = 7), working in confined workspaces (n = 5), worrying about work quality (n = 4), balancing work and personal responsibilities (n = 4) and working in cold temperatures (n = 4) resulting in exposure to biomechanical, psychosocial, physical and biological hazards. The psychosocial hazards associated with weaving were identified in many of the weaving activities such as heavy workloads, working at night, working at a fast pace, balancing work and other home responsibilities and factors related to working with the NGO. The NGO pieces utilize very fine wool and incorporate complex designs and finishing techniques that, in many cases, had to be newly learned by the weavers. Moreover, the strict quality control standards of the NGO left many participants concerned that their work could be rejected, resulting in re-doing the piece or having to re-pay the cost of the wool used to make the piece.

**Table 2:** Work hazards and sources of exposure.

Work Type and Activities	Hazard Type	Source of Exposure
**Weaving (n = 10)** • Weaving for long hours without breaks (8) • Weaving at night/poor lighting (7) • Weaving in a small, confined workspace (5) • Worrying about the quality of one’s own work (4) • Balancing work and personal responsibilities (4) • Working in cold temperatures (4) • Inhaling wool dust (3) • Weaving complex designs and finishing pieces (3) • Assembling the loom (2) • Weaving at a fast pace (1)	• Biomechanical • Psychosocial • Physical • Biological	• Heavy workload • Awkward postures • Repetitive movements • Stress • Poor lighting • Wool dust • Working fast
**Wool Production (n = 8)** • Spinning wool (2) • Dyeing wool (2) • Dyeing wool (chemical inhalation) (3) • Cleaning wool (1)	• Biomechanical • Chemical	• Awkward postures • Repetitive movements • Heavy load • Chemical inhalation
**Housework (n = 3)** • Cleaning (3) • Doing laundry (2) • Cooking (3)	Biomechanical	• Awkward postures • Repetitive movements
**Farm work (n = 3)** • Planting vegetables and fruit (3) • Taking care of livestock (3)	Biomechanical	• Awkward postures • Heavy loads

Among non-weaving work, participants shared that housework (n = 3), including cleaning, doing laundry and cooking, along with farm work (n = 3) resulted in awkward postures, repetitive movements and heavy lifting, or biomechanical hazards. Given the home-based nature of the work, participants shared how non-weaving hazards had direct effects on weaving tasks. For example, participants (n = 3) discussed experiencing back pain from being hunched over doing farm work for long periods or doing housework (e.g. cleaning, cooking) that required lifting, repetitive movements and awkward postures. While participants understood that these health issues were exacerbated by carrying out other work with similar hazards (including weaving), they also expressed the need to work through these health issues regardless of the pain.

## Discussion

Our sample of rural, home-based Mapuche weavers reflected many of the prevalent demographic characteristics of the craft sector, namely Indigenous and rural populations with limited educational and economic opportunities. The weavers we spoke to shared how their weaving work allowed them not only to support their families, but also to create economic independence and to establish themselves as financial decision makers within their families. They expressed pride in being able to financially support their children’s educational aspirations, but also stressed the importance of younger generations learning Mapuche weaving techniques.

Carrying out weaving and non-weaving work at home exposed participants to a range of hazards and health issues supported in the literature and reflective of the experiences of informal and home-based workers. These issues directly speak to the SDG 8 “Promote sustained, inclusive and sustainable economic growth, full and productive employment and decent work for all” which recognizes the need to address informal workers in a standard way to protect the health and safety and employment rights of these workers [40]. A focus of this research was assessment of home-based workspaces in the artisans’ own words and suggested improvements that would directly impact their health. The majority of weavers interviewed used common area workspaces which they described as cramped and requiring dismantling throughout the day to accommodate other domestic tasks. While two weavers had dedicated workspaces that allowed them to focus exclusively on their weaving work, these spaces were also so poorly insulated that they were uncomfortable and unsafe to use during the winter months. One of the major strengths of this study was collaborating with the weavers to identify and implement ergonomic solutions that enhanced their work environment and improved their overall health.

Aside from these more traditionally described occupational hazards, there is a growing appreciation of the impact of “work organization” on worker health and well-being [60, 61]. Mapuche weavers and other home-based artisans have irregular seasonal and even daily work schedules. They are generally treated as independent contractors in labor laws, but they are also informal workers, making them difficult to capture and support in terms of employment rights and mandated occupational safety and health protections. Employer based insurance (health, unemployment, paid sick leave, vacation time) is not provided in this work arrangement. Finally, “piece work” (being paid by the piece rather than by the hour) has been repeatedly identified as an unfair system of compensation for work and one that was expressed by participants in this investigation [62]. Upon completion of the study, the NGO worked with one of the investigators to interview staff and weavers about the possibility of creating a non-profit arm of the organization. The non-profit arm would be overseen by the weavers, providing them with greater input into the design and decision-making processes, while also creating additional work opportunities (e.g. educational workshops) for those weavers who wanted to transition from or reduce their weaving workload.

## Limitations

This study used purposive sampling to obtain a representative sample of the weavers that work with the NGO. Given the small sample size and the sampling procedure, these findings may not be generalizable to all home-based rural weavers or weavers that live in more urban areas, do not weave at home and do not work for an organization. Collaborations between artisans and designers are becoming more common as interest in traditional fair-trade and sustainable crafts have increased in the global market [63, 64]. These collaborations have proven successful at building on traditional techniques, increasing their marketability (e.g. meeting quality control standards) and providing greater and more sustainable economic opportunities for artisans [63–65]. These types of partnerships are increasing at the global scale as more consumers become better aware of the artisan market linkages. Approaches to investigating these relationships and their impact on the health and well-being of Indigenous workers is of critical importance.

The nature of the contextual interviews also introduced the “observer effect,” wherein the presence and participation of the investigators may have influenced participants’ behaviors during the observations or responses during the interviews. In qualitative research, the role of the investigator as an instrument of the research—both in execution and interpretation—must be taken into account.

In developing the time-wheel, the investigators relied on self-reports from the participants with regards to daily activities and time devoted to each activity, introducing recall bias. Additionally, the interviews took place during the fall season and therefore discussions about summer, spring and winter activities were also subject to recall errors. Due to the remote locations of participants’ homes and transportation constraints, the investigators were unable to observe participants working early in the morning and late in the evening and, therefore, relied on participants’ responses for these periods. The investigators identified this limitation early on in the study and were able to observe three weavers working in the evening as part of initial phase of the study. As part of these observations, weavers were asked to demonstrate what adjustments, if any, they made to their workspace and/or their techniques. The investigators also observed participants weaving to identify adjustments and hazards and asked participants about the types of health issues they experience while weaving in the evening. Weavers also provided estimates and ranges of time spent weaving in the evening. The investigators summarized this information and presented it to a subset of the weavers to confirm these findings were applicable to them as well. The NGO staff introduced the project to the weavers and identified potential participants for the interviews. As part of the informed consent process, investigators stated that participants’ responses would remain confidential, however, the NGO’s presence throughout the project may have influenced participants’ behavior and responses

## Conclusion

The craft sector’s workforce and annual revenue are projected to grow, with revenue expected to reach $1 trillion by 2024 [32]. Increased attention to workers’ health and work environment are needed to ensure the sustainability of this sector. As demonstrated in this study, contextual interviews coupled with participatory approaches provide a more holistic understanding of the health of home-based workers. In addition, these approaches generate solutions with the potential to improve the health, well-being and longevity of this workforce.

## Supporting information

S1 File English-language interview guide. (PDF)

S2 File Spanish-language interview guide. (PDF)

S3 File Contextual interviews codebook. (PDF)

S4 File English consent form. (PDF)

S5 File Spanish consent form. (PDF)
