# Supplementary material for: A day in the life: Using contextual interviews to understand the health of home-based Mapuche weavers
Source: PLOS Glob Public Health. 2022 May 10;2(5):e0000353. doi: 10.1371/journal.pgph.0000353 (PMC10021899; doi:10.1371/journal.pgph.0000353)
Supplement: S1 File — (PDF) [file pgph.0000353.s001.pdf]

S1. English-language Interview Guide

General:

1. Tell me about yourself

Weaving Background:

2. How old were you when you learned how to weave?

3. How long have you been weaving, either as a hobby or as a job?

Workday Organization:

4. Tell me about your workday?
   Prompts:
   - What do you do to prepare for your workday?
   - What time do you start?
   - What do you do during a typical workday?
   - How do you arrange your workday (work only mornings?, only evenings?, throughout the day?)
   - When do you take breaks?

Weaving Work

5. Please describe how you made this piece. (Point to a textile that the weaver is working on or has finished)
   Prompts:
   - How long did it take you to make this piece?
   - Did you dye the wool (natural or chemical)?
   - What kinds of techniques were involved?
   - Did you work with anyone else on this piece?

6. What type of weaving do you do or do you specialize in?
   Prompts:
   - What types of techniques do you use? (straight or with design)
   - Do you embroider?
   - Do you work on specific garment pieces?

Workspace:

7. Tell me about the area where you do your weaving work?
   Prompts:
   - How would you describe your workspace?
   - In what area(s) of the house do you weave?
   - Is it a comfortable place to work? Do you feel comfortable working there?
   - Is there anything you would change about your workspace?

8. Please tell me about your weaving tools
   Prompts:
   - How long have you had your loom?
   - What kind of tools do you use?
   - Is there anything you would change about your loom? Weaving tools?

Other Work/Household Activities:

9. What other work or tasks do you do (not related to weaving) during a typical day?
   - Please describe this work or tasks
   - During a typical day, how much time do you spend doing this work or task?
   - Does this work or task cause you any discomfort or pain?

10. Please tell me about the work you do around the house (not related to weaving).
    Prompts:
    - What type of housework do you do (clean, cook meals, care for children, etc.)?
    - During a typical day, how much time do you spend doing housework?
    - Does this work or task cause you any discomfort or pain?

Health:

11. In a typical day, do you experience any pain or discomfort?

    Prompts:
    - Can you describe the pain or discomfort?
    - How long have you experienced this pain or discomfort?
    - What do you think causes this pain or discomfort?
    - Do you ever feel any pain or discomfort when you're weaving?
    - Do you ever feel any pain or discomfort when you're doing other work (in or out of the house)?
