# Supplementary material for: A day in the life: Using contextual interviews to understand the health of home-based Mapuche weavers
Source: PLOS Glob Public Health. 2022 May 10;2(5):e0000353. doi: 10.1371/journal.pgph.0000353 (PMC10021899; doi:10.1371/journal.pgph.0000353)
Supplement: S2 File — (PDF) [file pgph.0000353.s002.pdf]

S2. Spanish-language Interview Guide

General:

1. Cuéntame de usted

Antecedentes de Tejer:

2. ¿Qué edad tenía cuando aprendió a tejer?

3. ¿Cuánto tiempo ha estado tejiendo, ya sea como un pasatiempo o como un trabajo?

Organización del Día Laboral:

4. Cuéntame de su día laboral.

   Solicitas:
   - ¿Qué hace para prepararse para su día de trabajo?
   - ¿A qué hora empieza?
   - ¿Qué hace durante un día típico de trabajo?
   - ¿Como organiza su día de trabajo? (¿trabaja solo en las mañanas? ¿Solo en las tardes? ¿Durante el día?)
   - ¿Cuándo toma descansos?

Trabajo de Tejer

5. Por favor describe como hico esta pieza. (Señale a un textil que la tejedora está trabajando o ha terminado).

   Solicitas:
   - ¿Cuánto tiempo le tomo en hacer esta pieza?
   - ¿Tiño la lana? (natural o química)
   - ¿Qué tipos de técnicas estuvieron involucrados?
   - ¿Trabajó con alguien más en esta pieza?

6. ¿Qué tipo de tejido haces o te especializas en?

   Solicitas:
   - ¿Qué tipos de técnicas usas? (liso o con diseño)
   - ¿Borda?
   - ¿Trabaja usted en piezas de prendas de vestir específicas?

Espacio de Trabajo:

7. Cuéntame del espacio en donde tejer.

   Solicitas:
   - ¿Cómo describiría su espacio de trabajo?

- ¿En qué área(s) de la casa tejes?
- ¿Es un lugar cómodo para trabajar? ¿Se siente cómoda trabajando allí?
- ¿Hay algo que cambiarías de tu espacio de trabajo?

8. Por favor cuéntame de sus herramientas de tejer

   Solicitas:
   - ¿Cuánto tiempo ha tenido su telar?
   - ¿Qué tipo de herramientas usa?
   - ¿Hay algo que cambiarías de tu telar? ¿herramientas de tejer?

Otros Trabajo / Actividades del hogar:

9. ¿Qué otros trabajos o tareas hacen usted (no relacionado con tejido) durante un día típico?

   Solicitas:
   - Por favor describe este trabajo o tareas
   - Durante un día típico, ¿cuánto tiempo pasa haciendo este trabajo o tarea?
   - ¿Le causa molestia o dolor este trabajo?

10. Por favor cuéntame del trabajo que hace en la casa (no relacionado con tejiendo).

    Solicitas:
    - ¿Qué tipo de tareas domésticas hace (limpiar, cocinar, cuidar a los niños, etc.)?
    - Durante un día típico, ¿cuánto tiempo pasa haciendo las tareas domésticas?
    - ¿Le causa molestia o dolor este trabajo?

Salud:

11. En un día típico, ¿experimenta algún dolor o molestia?

   Solicitas:
   - ¿Puede describir el dolor o molestia?
   - ¿Cuánto tiempo ha experimentado este dolor o molestia?
   - ¿Qué piensas que causa este dolor o molestia?
   - ¿Siente algún dolor o molestia cuando está tejiendo?
   - ¿Siente algún dolor o molestia cuando estás haciendo otro trabajo (dentro o fuera de la casa)?
