# Supplementary material for: A day in the life: Using contextual interviews to understand the health of home-based Mapuche weavers
Source: PLOS Glob Public Health. 2022 May 10;2(5):e0000353. doi: 10.1371/journal.pgph.0000353 (PMC10021899; doi:10.1371/journal.pgph.0000353)
Supplement: S3 File — (PDF) [file pgph.0000353.s003.pdf]

Contextual Interviews Codebook

General Coding Rules:

- Memo any quotations, thoughts, observations you think are important to revisit
- Do not code “no” responses
- Double code health issue with health cause or health treatment as much as possible
- Double code Seasonal Work with Work Type
- Prioritize Education_Weaving over Support Sources
- Support Sources should be coded only if they directly contribute to weaving work
- Prioritze SuggestedChanges over WorkType_
- Double code Seasonal Change with WorkType_
- Prioritize IDHealth_Cause over Worktype
- Prioritize WorkType_House over WorkType_Children
- Motivation needs to be directly stated by the weaver

Family	Code	Atlas Code	Definition	Inclusion or Exclusion Criteria	Example
Work Type	Farm Work	WorkType_ Farm	• Farmwork (seasonal) being done on the weaver's land (or land they rent);<br>• Growing crops, vegetables, fruits;<br>• Feeding and caring for animals<br>• looking for wood	**Include:**<br>• current work<br><br>**Exclude:**<br>• Discussion of skills or work done in the past	“Y también trabajo en el campo para pero eso es para el consumo de mi mi familia también.”
	Caring for Children	WorkType _Children		**Include:**<br>• current work	“Y de ahí ella, ella se levantan y todo y ya despue eh, comienzo con el, con mi

Family	Code	Atlas Code	Definition	Inclusion or Exclusion Criteria	Example
				**Exclude:**<br>• Discussion of skills or work done in the past	hijo también al allá a despertarlo y a lavarlo y prepararlo porque ya a las ocho y media lo vienen a buscar a el.”
	Housework	WorkType_House	• Cleaning the house<br>• Cooking<br>• Doing dishes<br>• Laundry<br>• Making a fire	**Include:**<br>• Current work<br>• Prioritize WorkType_House over WorkType_Children<br>**Exclude:**<br>• Discussion of skills or work done in the past	“Si como a las 9 casi siempre porque me hago todo lo que aseo, paso a lavar el baño y todo y de ahí hago fueguito, limpio la cocina, baro.”
	Other Work	WorkType_Other		**Include:**<br>• Current work<br><br>**Exclude:**<br>• Discussion of skills or work done in the past	“Así que eh es mas eh encuentro que es ma, es ma relajado, ma voy hacer eh una cosa especifica, tengo claro lo que voy, (inaudible) tengo que hacer la muestras pero eh es lo que es lo que me gusta hacer…

Family	Code	Atlas Code	Definition	Inclusion or Exclusion Criteria	Example
					Y y se que que mi tiempo va estar completamente dedicado a eso no mas.
	Weaving Textiles	WorkType _Weaving	• Work with NGO, Chol-Chol, other cooperatives or organizations<br>• Work of their own they sell	**Include:**<br>• Current work<br><br>**Exclude:**<br>• Discussion of skills or work done in the past	
	Knitting	WorkType _Knitting		**Include:**<br>• Current work<br><br>**Exclude:**<br>• Discussion of skills or work done in the past	"Claro. Entonce eso es lo que hay que el el trabajo que uno tiene que tener y llevar bien el palillo para que no le quede tan desparejo. Claro porque si uno suelta un poquito el palillo y el otro junto lo deja mas apretado le va quedando desparejo y ese es el cuidado que hay que eh que hay que tener."
	Natural Dyeing	WorkType _Dyeing	• The steps involved in the natural dyeing process include: looking for raw materials (leaves, flowers, vegetables, etc); boiling,	**Include:**<br>• If any of the steps are identified, code as	"Si. Ya en la tarde ya si tengo lana para teñir, ya me dedico a preparar la lana, después 12."

Family	Code	Atlas Code	Definition	Inclusion or Exclusion Criteria	Example
			washing and drying the wool; adding a mordant	natural dyeing<br>• current work<br><br>**Exclude:**<br>• Discussion of skills or work done in the past	
	Wool Production	WorkType _WoolProd	• The steps involved in the wool production process include: shearing the lamb, washing and drying the wool, spinning and twisting the wool	**Include:**<br>• If any of the steps are identified, code as wool production<br>• current work<br><br>**Exclude:**<br>• Discussion of skills or work done in the past	Entrevistadora: ¿Y sienta algún dolor o molestia cuando esta trabajando en esto, tenido?<br><br>"Eh, el humo no ma, el ojo…Porque el humo anda así cuando esta muy abierto ahí, po."
	Splitting Wool for NGO	WorkType _Splitting		**Include:**<br>• Current work	"Como las 8, 8:30, ma o meno, si de repente me quede pillando hilo lo que sea yo, partiendo hilo, asi,

Family	Code	Atlas Code	Definition	Inclusion or Exclusion Criteria	Example
				**Exclude:**<br>• Discussion of skills or work done in the past	abriendo. Y de ahí cuando tengo el tejido, tengo, por ejemplo, (inaudible) eh, abro el hilo, po, parto hilo”
	Weaving Teaching	WorkType _WeaveTeach		**Include:**<br>• Current work<br><br>**Exclude:**<br>• Discussion of skills or work done in the past	“Si. Y también ha sido monitora en el colegio. También estuve un tiempo ensenándole a los niños a hacer el urdido, a tejer. También estuve ahí con los niños trabajando un tiempo a través de la agrupación Nehuen (sp). Nehuen (sp) tiene un, trabaja con los niño y ahí ahí anos que trabajan en telar, la ensenan también el tema de los, do lo que la greda. También han trabajado con greda ellos.”
	Community Obligations	WorkType _Community	• Involvement in associations, cooperatives, workshops, etc. in the community	**Inclusion:**<br>• Weaving workshops<br>• Capacity building workshops (cooking, managing a	Entrevistadora: ¿Y que tipo de capitación-capacitaciones?<br><br>“Eh, para mejorar el negocio. Para aprender a a negociar, a sacar las cuentas, a separar lo que es dinero, lo que es para el

Family	Code	Atlas Code	Definition	Inclusion or Exclusion Criteria	Example
				business, etc.)<br>• Meetings to discuss improving the community or addressing issues<br>• Gatherings in the commmunity for celebrations, funerals<br>• current work<br><br>**Exclude:** Discussion of skills or work done in the past	gasto y lo otro que el capital que tiene que dar para seguir trabajando."
	Sales	WorkType_ Sales	• The sale of any products including: textiles, produce, AVON, baked goods, etc.	**Include:**<br>• Current Work<br><br>**Exclude:**	

Family	Code	Atlas Code	Definition	Inclusion or Exclusion Criteria	Example
				• Work done in the past	
N/A	Work Seasonal Changes	WorkChange_Seasonal	• Captures changes in work in the Spring and Summer		“Como que siempre el el día el tiempo esta así, si. Porque tiene que ser, eh en el verano, por ejemplo, ya ahí dedica o un día por eh todo el día en el campo. Pero yo siempre estoy tejiendo, siempre tengo trabajo y y tengo fecha que cumplir así que tengo que estar con bien conectada con mi tejido”
Weaving Motivation	Economic	Motiv_Economic	• Weaving as a form of income	Inclusion: • Needs to be explicitly stated by the weaver	“Eh, si. Y y y me quedo eso no me olvide nunca de eso y y siempre dije ‘como le puedo ayudar? ¿Como lo va podemos hacer para ayudarle?’ así que el momento que yo tuve oportunidad de ayudar, ayude a tejer, a limpiar la lana ayudar a lavar, ayudar ayudar siempre ayudar… Si. Eso fue eh eso fue la razón que yo dije esto será muy difícil pero tengo que aprenderlo, tengo que

Family	Code	Atlas Code	Definition	Inclusion or Exclusion Criteria	Example
					aprenderlo.”
	Allow to Stay Home	Motiv_Home	• Choosing to weave because it allows the weaver to work from home	Inclusion:<br>• Needs to be explicitly stated by the weaver	“Trabajar en otra cosa pero antes quiero trabajar porque estoy en mi casita. “<br><br>Entrevistadora: ¿Y es, eso es importante para usted?<br><br>“Muy importante porque no dejo a lado de mi familia, mi marido, mi hijo que me queda soltero todavía, mis nieto, tengo y no es un trabajo obligado si, po. Uno le pone fecha y se apure y si quiere mas mas plata, trabaja mas, po.”
	Independence	Motiv_Indep	• Choosing to weave in order to become more independent (financial, personal, etc)	Inclusion:<br>• Needs to be explicitly stated by the weaver	
	Keep Tradition	Motiv_Tradition	• Choosing to weave to maintain the Mapuche tradition or other weaving techniques	Inclusion:<br>• Needs to be explicitly	

Family	Code	Atlas Code	Definition	Inclusion or Exclusion Criteria	Example
				stated by the weaver	
	For use in Home	Motiv_Usehome	• Weaving products for self-sustainability (use in the home)	Inclusion:<br>• Needs to be explicitly stated by the weaver	
	Likes to Weave	Motiv_likes		Inclusion:<br>• Needs to be explicitly stated by the weaver	
N/A	Weaving Education	Education_Weaving	• Who the weaver identifies as having taught her how to weave.<br>• Any skills, techniques or practices related to weaving	**Inclusion:**<br>• Family member<br>• Workshop<br>• Self taught<br>• other weavers<br>• Skills developed<br>• Techniques<br>• Wool production<br>• Natural dyeing<br>• Prioritize Education_Weaving	Entrevistadora: um, quería empezar preguntándole que le ¿quien le enseno a tejer o como a empezó a tejer?<br><br>"Acá mi mama era tejedora… Si. Ella era tejedora y y como le contaba el otro día que ella quedo viuda muy joven. Nosotro éramos chicos y y ella la la forma de sacar adelante a su familia eh fue con los tejido. "

Family	Code	Atlas Code	Definition	Inclusion or Exclusion Criteria	Example
				over Support Sources	
N/A	Weaving Workspace	Workspace _Weaving	• Where the weaver works and any comments related to the workspace	**Inclusion:**<br>• Dedicated weaving workspace<br>• Living Room<br>• Dining Room<br>• Outside<br>• Other	"Es que mas fácil uno tiene su tejido ahí llega y sentar a tejer no ma, po. En cambio casi si había visita no podía tejer tiene porque mi palo estorbaban había que guardar el por los dia que la visita están aca"
Hazards	Biomechani cal	Hazards_Bi omech	• Biomechanical injuries occur when the forces on a body tissue (e.g. muscle, tendon, ligament, bone) are greater than the tissue can withstand. These injuries can occur suddenly as a consequence of a single exposure to a high force; they can also arise gradually, as a consequence of repeated or long-duration exposure to lower levels of force.	**Inclusion:** -<br>• Using machinery or tools (shovel, ax, picks)<br>• Lifting heavy equipment (loom)<br>• Bending over for long periods<br>• Kneeling<br>• awkward postures	Entrevistadora: ¿Y en ese momento cuando esta haciendo algo le tiene algún dolor o molestia?<br><br>"Eh, si la espalda…Si. Las cadera, los hueso, donde hace fuerza uno.<br><br>Entrevistadora: ¿La fuerza empieza cuando que? ¿Cuando esta haciendo que?<br><br>"Si, cuando estamos haciendo, ejemplo, ya pa

Family	Code	Atlas Code	Definition	Inclusion or Exclusion Criteria	Example
				• Sitting for long periods	tirar el agua, pa, hay veces no están los hombre en la casa y hay que hacerlo uno.”
	Biological	Hazards_Bio	• These exist in exposures to bacteria, viruses, fungi and other living organisms that can cause acute and chronic infections by entering the body either directly or through breaks in the skin.	**Inclusion: -**<br>• Working with animals<br>• working with plants<br>• preparing wool<br><br>**Exclusion:**<br>• Dealing with treated wool	“Si. Que tengo uno centímetros de lana pues no queda muy cortita, cuesta para para hilarla. Claro. Entonce ahí se esquila y después se leva la lana se escoge la la mejor lana, se lava,”<br><br>Entrevistadora: ¿Y usted esquila?<br><br>“Y ahí después se lava, se seca y si una la quiere teñir así en en lana no mas uno lo tiñe la mande hilar así tenida.”
	Chemical	Hazards_Chem	• Harmful chemical compounds in the form of solids, liquids, gases, mists, dusts, fumes and vapours exert toxic effects by inhalation (breathing), absorption (through direct contact with the skin) or ingestion (eating or drinking)	**Inclusion:**<br>• wood fumes<br>• wool dust<br>• natural or chemical dyes<br>• dyeing	Entrevistadora: ¿Se pone algo como mordaz o algo que?<br><br>“Si.”<br><br>Entrevistadora: Para que

Family	Code	Atlas Code	Definition	Inclusion or Exclusion Criteria	Example
				• fumes<br>• chemical solvents	"Eh, depende el color que uno quiera, eh, piedra lumbre, sal, si."
	Physical	Hazards_Phys	• These include excessive levels of noise, vibration, illumination and temperature,	**Inclusion:**<br>• Noise<br>• Lighting<br>• Temperature	Entrevistadora ¿Bueno, estas cómodo allá?<br><br>"Eh, cómoda si pero a vece muy entumida, po. Mucho frio hace…Si. Igual a veces el espacio se hace chico porque la bodega en donde guardamos las herramienta de trabajo del campo lo lo saco, lo ha que cosechamos, lo guardamos allá. Y cuando no tenemo campo arrendado esa bodega la tenemos tenemos que guardar el forraje para el invierno los animales y esta lleno."
	Psychosocial	Hazards_Psychosocial	• A psychological hazard is any hazard that affects the mental well-being or mental health of the worker and may have physical effects by overwhelming the individual coping mechanisms and impacting the workers ability to	**Inclusion:**<br>• Stress<br>• Repetitive tasks<br>• Low pay<br>• Lack of recognition	"Pero cuando, por ejemplo, cuando uno le, por ejemplo, cuando uno le, le duele la cabeza igual el animo esta mas bajo esta mas bajo avanza meno en todo lo que hace avanza meno."

Family	Code	Atlas Code	Definition	Inclusion or Exclusion Criteria	Example
			work in a healthy and safe manner.	• Long work hours<br>• fatigue	
Health-Identified Health Issues	Arm Pain	IDHealth_Arm	• Identified by the weaver (or an affirmative response if the interviewer asks).		"Si. ¿Que mas? Ayer estuvi lavando y repente meto la mano al agua helada y despue tenia un dolor que no podía tejer y me heche cremas (inaudible) y me paso."
	Back Pain	IDHealth_Back	See above	**Inclusion:**<br>• Back or waist pain	"Si. Eh, cuando hago el lo que es aseo general así me toca todo el día a veces hacer como un asea completo ahí también siento molestia en la espalda."
	Depression	IDHealth_Depression	See above	**Exclusion:**<br>• Fatigue	"Eh, ese un es algo que a uno no se es como una también puede ser, es como una terapia para uno que uno sufre que se yo un poco de depresión que y ahí un grupo uno tontea, usted sabe, a mi me gusta tontearme me gusta, reírme, me gusta echar talla cuando estoy con gente por eso."

Family	Code	Atlas Code	Definition	Inclusion or Exclusion Criteria	Example
	Difficulty Breathing	IDHealth_DiffBreathe	See above	**Inclusion:**<br>• Tightness in chest<br>• Sneezing<br>• coughing	“Claro porque el tema del hi-del del de las pelusitas del del hilo de la lana de de alpaca también me empieza como a molestar aquí entonces por eso me coloco la mascara cuando tejo.”
	Eyesight Issues	IDHealth_Eyesight	See above		Entrevistadora: ¿Y me había contado cuando le hecimos la encuesta que uh tenia alguna problema con su vista?<br><br>“Si po, eh que estoy corto de vista para en entrar aguja así, igual cuando esta muy oscura la no no me doy a tejer me queda caballito atrás, po.”
	Finger Pain	IDHealth_Finger	See above		“Yo cargo este dedo, este (muestra el dedo gordo). Porque aquí donde tengo el tengo aquí como como un nervio que pienso que yo que algo que me”
	Hand Pain	IDHealth_Hand	See above		Entrevistadora: ¿Y cuando dijiste, eh, por la tema la terapia, ehm, en que en que sentido?

Family	Code	Atlas Code	Definition	Inclusion or Exclusion Criteria	Example
					“Eh, de mis mano. Para tener actividad en mis mano, en mis hueso, de la de la mano porque donde muevo las mano así me, yo pienso que como una terapia porque una esta en movimiento.”
	Headache	IDHealth_Headache	See above		“Eh, si yo creo que si porque cuando uno esta bien bien esta esta bien haciendo todo y bien, po. Pero cuando, por ejemplo, cuando uno le, por ejemplo, cuando uno le, le duele la cabeza igual el animo esta mas bajo esta mas bajo avanza meno en todo lo que hace avanza meno.”
	Neck Pain	IDHealth_Neck	See above		
	Other Pain	IDHealth_Other	See above		
	Shoulder Pain	IDHealth_Shoulder	See above		Entrevistadora: ¿Cuando le da ese dolor en su hombro?<br><br>“Ese me da eh estuve un tiempo con bien seguido me daba el dolor. Pero

Family	Code	Atlas Code	Definition	Inclusion or Exclusion Criteria	Example
					ahora ya hace como dos meses que no no no me da el dolor, si.”
	Wrist Pain	IDHealth_Wrist	See above		“Solamente la mana pero me parece que eso del cambio de agua temperature”<br><br>Entrevistadora: Ah, ya. ¿La muñeca?<br><br>“Si.”
	Chronic Pain	IDHealth_Chronic	See above	Inclusion:<br>• Weaver needs to explicitly state “chronic” to use this code	“Mi enfermedad es eh, tres meses que me opere no me encuentro bien eh, me operación y lo otro que mi de mis hueso que vivo enferma también, po. Con dolores de hueso, mis mano, dolor de cabeza, que sufro cefalea tensional”
Health-Identified Causes	Age	IDCause_Age	Identified by the weaver (or an affirmative response if the interviewer asks)	Inclusion:<br>• Prioritize IDHealth_Cause over Worktype	
	Farm Work	IDCause_Farmwork	See above	Inclusion:	“Eh ahí duele la espalda. Hacemos eso, por ejemplo,

Family	Code	Atlas Code	Definition	Inclusion or Exclusion Criteria	Example
				• Prioritize IDHealth_Cause over Worktype	cuando hay que picar la huerta igual también eh la espalda.”
	Housework	IDCause_Housework	See above	Inclusion:<br>• Prioritize IDHealth_Cause over Worktype	“Si. Eh, cuando hago el lo que es aseo general así me toca todo el día a veces hacer como un asea completo ahí también siento molestia en la espalda.”
	Natural Dyeing	IDCause_NatDye	See above<br>• The steps involved in the dyeing process include: looking for raw materials (leaves, flowers, vegetables, etc); boiling, washing and drying the wool; adding a mordant	**Inclusion:**<br>• If any of the steps are identified, code as natural dyeing<br>• Prioritize IDHealth_Cause over Worktype	Entrevistadora: ¿Y sienta algún dolor o molestia cuando esta trabajando en esto, tenido?<br><br>“Eh, el humo no ma, el ojo… Porque el humo anda así cuando esta muy abierto ahí, po.”
	Other Health Issue	IDCause_OtherHealth	See above		“Es que yo tengo artrosis en todo el cuerpo casi.”
	Weaving	IDCause_Weaving	See above	**Inclusion:**	“Esa la vista, po. Eh, la vista. El hilo negro, es la vista.”

Family	Code	Atlas Code	Definition	Inclusion or Exclusion Criteria	Example
			• Weaving can include: setting up the warp, weaving, finishing the piece, sitting for long hours, cold/hot workspace, dust and allergens; working with wool	• If any of the steps are identified, code as weaving<br>• Prioritize IDHealth_Cause over Worktype	
	Wool Production	IDCause_WoolProd	• See above<br>• The steps involved in the wool production process include: shearing the lamb, washing and drying the wool, spinning and twisting the wool	**Inclusion:**<br>• If any of the steps are identified, code as wool production<br>• Prioritize IDHealth_Cause over Worktype	Entrevistadora: ¿Y usted cuando esta hilando siente algún dolor o molestia?<br><br>“Eh, la espalda…Si, eh, por eso hilo mas parada que sentada”
	Knitting	IDCause_Knitting	See above	Inclusion:<br>• Prioritize IDHealth_Ca	

Family	Code	Atlas Code	Definition	Inclusion or Exclusion Criteria	Example
				use over Worktype	
Health-Treatment		ID_Treatment		**Inclusion:**<br>• Breaks<br>• Cream<br>• Exercise<br>• Aspirin<br>• Tea<br>• Prescribed Medicine<br>• Modified Work<br>• No treatment	"Si. Porque cuando no que como uno esta tejiendo esta como así y como que se mueve para allá, paca entonces ya ahí me siento un ratito y me acomodo así y de hay se me calma el el dolor y ahí sigo tejiendo de nueve y de ahí vuelve el dolor y ahí calmo y sigo tejiendo."
Health-Barriers to Treatment	Administrative	BarriersTrt_Admin		**Inclusion:**<br>• long appointment wait<br>• insurance type	"Eh, es que lo que pasa es que la la operación tienen que darme una hora y yo fui y me dijeron que porque mi iban dicho que fuera en un ano a pedir la hora…Y fui y me dijeron que no que tenia que hablar con otro medico que para que me diera una una hoja, eh, una consulta y con ese para que me me den la hora."<br><br>Entrevistadora: ¿Tuvo que, tuvo que esperar una ano

Family	Code	Atlas Code	Definition	Inclusion or Exclusion Criteria	Example
					para ir a pedir?<br><br>"Si"
	Medicine that Doesn't Work	BarriersTrt_MedsNotWork			"No. Porque no quitan los dolores paracetamol."
	Chronic-No Treatment	BarriersTrt_ChronNoTrt			"Si. Pero para la artrosis no hay remedio entonce así que dan paracetamol no ma[s]."
	Access to Healthcare	BarriersTrt_Access			
	Other	BarriersTrt_Other			
N/A	Suggested Changes or Improvements	Suggest_Changes		**Inclusion:**<br>• Dedicated workspace<br>• Better Lighting<br>• Just Payment<br>• Transportation<br>• Weaver Orientation<br>• Other	"Si. Pero si que unos paño largo que hice eso lo urdí afuera y despue le arrollamos con la Marisol me ayudaba y los traía pa dentro. Eso me gustaría igual tener una piecita así que eso me encantaría tener una piecita donde tener mi tejido porque igual po si voy hacer (inaudible) tengo que arrolladar mi tejido acá así que me queda poquito tengo que

Family	Code	Atlas Code	Definition	Inclusion or Exclusion Criteria	Example
					envolverlo bien en una sabana.”
NGO	Background Info	NGO_Background	Information relating to NGO's history	**Inclusion:**<br>• How NGO started<br>• Founding members<br>• Initial Workshop<br>• Convention	“Si. Si y después ya cuando volvió ya ahí me llamo y me dijo que que en realidad quería hacer eh, y trabajar y que y que que que pensaba yo, po. Yo le dije que me parecía muy bien que me parecía muy bien y desde un comenzó le dije que eh, si que si se va trabajar así que se trabajara en un comienzo con las mejore tejedora con las mejore y y que yo creía que si eso (inaudible). Y así que ahí empe-empezemo mucha capacitaciones, muchos tallere. Iba a lunes, miércoles y viernes (reir) y ahí igual yo en eso tiempo vivía acá con mi mama y ella no veía así que tenia que coordinar mucho muy bien en mi mi cosa mis compromiso con mi hermano.”

Family	Code	Atlas Code	Definition	Inclusion or Exclusion Criteria	Example
NGO	Practices	NGOPractices	• Any practices or processes identified by the weaver that relate to NGO.<br>• Prioritize NGO Practices over WorkType_Weaving	**Inclusion:**<br>• Onboardin<br>• Giving the weavers a sample project<br>• Providing Wool<br>• Payment<br>• Spec Sheet<br>• Splitting Wool<br>• Working with different types of wool<br>• Quality Control	"Si, después la Señorita X me dio una chaquetita. Unas tres piezas. Y no me podían dar mas porque no me conocían y como tejía. Si me quedaba bueno, me dijo yo le voy a dar mas tejido y si no, no. Y me quedo bueno y me dio otro, me dio otro mas dos. Después me dio tres, después me dio cinco y así fue aumentando yo solita."
NGO	Comments	NGOComments	• Comments (positive and negative) made by the weavers regarding NGO	**Inclusion:**<br>• Positive and negative comments<br>• Uncertainty about the future<br>• Relationship with NGO	"Ojala, Dios quiera, le valla bien muy bien a la Señorita X que no no los falte trabajo a nosotro la gente campesina. Porque es un ingreso muy lindo y de una alegría muy grande casi no se puede decir como es la alegría porque uno están en

Family	Code	Atlas Code	Definition	Inclusion or Exclusion Criteria	Example
				management<br>• Comparison with other NGO weavers<br>• Founder vs. more recent weavers<br>• Comments on the quality of other weavers' work	su casa, no son, no han con reunión a cada rato muy comprensiva."
N/A	Support Sources	SupportSources	• Positive and Negative sources of support that directly impact weaving work	**Inclusion:**<br>• Positive and negative sources<br>• Family<br>• Friends<br>• Community<br>• Other weavers<br>• Organizations or cooperative<br>• Employer	"Termino el diseño, me para ahí porque cuando esta mi esposo igual hago el almuerzo mas tarde entonces igual eh, bueno con que este el a veces el me cocina igual, po. Me dice, 'yo voy a cocinar, tu teje no ma.'Así que el co-cocina y yo digo 'hay que bueno' porque a el le queda super rica la comida (reir)."
