# Supplementary material for: A day in the life: Using contextual interviews to understand the health of home-based Mapuche weavers
Source: PLOS Glob Public Health. 2022 May 10;2(5):e0000353. doi: 10.1371/journal.pgph.0000353 (PMC10021899; doi:10.1371/journal.pgph.0000353)
Supplement: S4 File — (PDF) [file pgph.0000353.s004.pdf]

APPROVAL
STARTS EXPIRES
03/03/2016 — 11/04/2016
UIC UNIVERSITY OF ILLINOIS AT CHICAGO
INSTITUTIONAL REVIEW BOARD

University of Illinois at Chicago
Research Information and Consent for Participation in Social Behavioral Research

Title: Developing an ergonomic intervention to decrease musculoskeletal disorders among home-based Mapuche weavers

You are being asked to participate in a research study. Researchers are required to provide a consent form such as this one to tell you about the research, to explain that taking part is voluntary, to describe the risks and benefits of participation, and to help you to make an informed decision. You should feel free to ask the researchers any questions you may have.

Principal Investigator Name and Title: Gabriela Gracia, MSc.

Department and Institution: Environmental and Occupational Health Sciences, University of Illinois at Chicago

Address and Contact Information: 2121 W. Taylor Chicago, IL 60612
ggraci2@uic.edu

Sponsor: National Institute of Occupational Safety and Health (NIOSH)

<u>Why am I being asked?</u>

You are being asked to be a subject in a research study about improving the health of weavers who use home-based workspaces.

You have been asked to participate in the research because you are a weaver who works with VOZ and are between the ages of 22 and 80.

Your participation in this research is voluntary. Your decision whether or not to participate will not affect your current or future dealings with the University of Illinois at Chicago. **If you decide to participate, you are free to withdraw at any time without affecting that relationship.**

Approximately 20 subjects may be involved in this research at UIC.

What is the purpose of this research?

Researchers are trying to learn more about home-based weavers along with weaving tasks, hazards and health risks. Researchers are also interested in learning about how to reduce musculoskeletal pain in your weaving work.

What procedures are involved?

This research will be performed at your home. We would like to observe the area where you do your weaving work two times and then ask you some questions. Each visit will take two to three hours.

The study procedures are

- Two site visits to observe the area where you do your weaving work. Each site visit will take about two hours. During this time, we will take photographs and video recordings of your weaving tasks and workspace. The video recordings will not capture any facial characteristics. You can choose not to have photographs and/or video taken and still participate in this study.
- One interview with you about your weaving work responsibilities, household responsibilities and your perceptions of health. This interview will take about two hours and will be audio recorded.

What are the potential risks and discomforts?

To the best of our knowledge, the things you will be doing have no more risk of harm than you would experience in everyday life. If any questions make you uncomfortable, you can choose not to answer them. If the photographs or video recordings make you uncomfortable you can choose not to have them taken. You may withdraw at any time.

Will I be told about new information that may affect my decision to participate?

During the course of the study, you will be informed of any significant new research information (either good or bad), such as changes in the risks or benefits resulting from participation in the research or new alternatives to participation, that might cause you to change your mind about continuing in the research. If new information is provided to you, your consent to continue participating in this research may be re-obtained.

Are there benefits to taking part in the research?

Taking part in this research study may not benefit you personally, but we [researchers] may learn new things that will help others.

What other options are there?

You have the option to not participate in this study.

What about privacy and confidentiality?

The people who will know that you are a research subject are members of the research team. Additionally, VOZ staff and anyone in your home during the workspace visits may be aware you are participating in a research project. Otherwise information about you will only be disclosed to others with your written permission, or if necessary to protect your rights or welfare (for example, when the UIC Office for the Protection of Research Subjects or State of Illinois auditors monitors the research or consent process) or if required by law. You will be assigned a unique identifier and your audio recording will be transferred to a software program on the PI's computer.

This study involves the audio recording of your interview with the researcher. Neither your name nor any other identifying information will be associated with the audio recordings or the transcript. Only the research team will be able to listen to the recordings. You will have the opportunity to review the audio recordings and transcripts

The research team will transcribe the recording. A transcript of your interview will be available 10 days after your interview. You will have 14 days to review your transcript. If you choose to review the transcript, you will have the opportunity to clarify information you have provided, request that certain quotes not be used, and withdraw consent entirely from the project. The transcripts and audio recordings will be destroyed upon completion of the study.

Study information that identifies you and the consent form signed by you will be looked at and/or copied for checking up on the research by: the National Institute of Occupational Health and Safety (NIOSH)

A possible risk of the research is that your participation in the research or information about you might become known to individuals outside the research. You will be assigned a unique identification number and any information linking you to the unique identifier will be kept on a separate password-protected file. Only the research team will have access to this information. Upon completion of the project, all data will be destroyed.

When the results of the research are published or discussed in conferences, no information will be included that would reveal your identity. The research team will have access to your audio interviews. You have the right to review the audiotapes of your interview and these tapes will be destroyed upon completion of the study.

What are the costs for participating in this research?

There are no costs to you for participating in this research.

Will I be reimbursed for any of my expenses or paid for my participation in this research?

You will receive $10.00 in cash for each completed study visit. If you do not finish the study, you will be compensated for the visits you have completed. If you complete the study, you will

receive a total of $20.00. You will receive your payment immediately after completing the site visits and the interview.

Can I withdraw or be removed from the study?

If you decide to participate, you are free to withdraw your consent and discontinue participation at any time.

Choosing not to be in this study or to stop being in this study will not result in any penalty to you or loss of benefits to which you are entitled. Specifically, your choice not to be in this study will not affect the present or future relationship with VOZ.

The Researchers also have the right to stop your participation in this study without your consent if they believe it is in your best interests.

In the event you withdraw or are asked to leave the study, you will still be compensated as described above.

Who should I contact if I have questions?
Contact the researchers Gabriela Gracia, MSc. at ggraci2@uic.edu or Dr. Hugo Romero at +56 45 22054 58 or hugo.romero@uct.cl:

- If you have any questions about this study or your part in it,
- If you have questions, concerns or complaints about the research.

What are my rights as a research subject?

If you feel you have not been treated according to the descriptions in this form, or if you have any questions about your rights as a research subject, including questions, concerns, complaints, or to offer input, you may e-mail the Office for the Protection of Research Subjects (OPRS) at uicirb@uic.edu or contact Dr. Hugo Romero from the Catholic University of Temuco at +56 45 22054 58 or hugo.romero@uct.cl or contact Jeanette Pérez Committee on Research Ethics, Catholic University of Temuco at: jeperez@uct.cl.

Remember:

Your participation in this research is voluntary. Your decision whether or not to participate will not affect your current or future relations with the University. If you decide to participate, you are free to withdraw at any time without affecting that relationship.

Understanding of Research:

I'm going to ask you a few questions about what you just read OR what we just reviewed.

- Name at least two things that will be expected of you during the study.
- Explain what you will do if you experience distress or discomfort during the study.
- Do you have to take part in this study or is it OK to say "no"?

- What is the purpose of this study?

Signature of Subject

I have read (or someone has read to me) the above information. I have been given an opportunity to ask questions and my questions have been answered to my satisfaction. I agree to participate in this research. I will be given a copy of this signed and dated form.

Your initials _________ indicate your permission to be photographed

Your initials _________ indicate your permission to be video recorded

______________________________ ____________
Signature Date

____
Printed Name

______________________________ ____________
Signature of Person Obtaining Consent Date (must be same as subject's)

____
Printed Name of Person Obtaining Consent
