# Supplementary material for: A day in the life: Using contextual interviews to understand the health of home-based Mapuche weavers
Source: PLOS Glob Public Health. 2022 May 10;2(5):e0000353. doi: 10.1371/journal.pgph.0000353 (PMC10021899; doi:10.1371/journal.pgph.0000353)
Supplement: S5 File — (PDF) [file pgph.0000353.s005.pdf]

APPROVAL
STARTS 03/03/2016 — EXPIRES 11/04/2016
UIC UNIVERSITY OF ILLINOIS AT CHICAGO
INSTITUTIONAL REVIEW BOARD

Universidad de Illinois en Chicago
Información y Consentimiento para Participación en el Estudio de Investigación de Conducta Social
Titulo: El desarrollo de una intervención ergonómica para disminuir los trastornos musculoesqueléticos en tejedoras Mapuche que trabajan en su hogar

Se le ha pedido participar en un estudio de investigación. Los investigadores estan obligados proveerle un formulario de consentimiento como este para explicarle lo que consiste el estudio de investigación, que participación es voluntaria, describir los riesgos y ventajas de participar, y ayudarle a tomar una decisión informada. Con confianza, consulte con los investigadores cualquier pregunta que tenga.

Nombre y Titulo del Investigador Principal: Gabriela Gracia, MSc.

Departamento y Institución: Ciencias de la Salud Ambiental y Ocupacional, Universidad de Illinois, Chicago

Domicilio e Información de Contacto: 2121 W. Taylor Chicago, IL 60612
ggraci2@uic.edu

Patrocinador: Instituto Nacional para la Seguridad y Salud Ocupacional (NIOSH)

¿Por qué se me pide participar?

Se le pide participar como sujeto en un estudio de investigación sobre el cuidado y las mejoras la salud de tejedoras que tejen en su hogar.

Se le pide participar como sujeto en un estudio de investigación porque usted es una tejedora que trabaja con VOZ y usted está entre las edades de 22 y 80.

Su participación en esta investigación es voluntaria. Su decisión de participar o no, no afectará sus relaciones actuales o futuras con la Universidad de Illinois en Chicago. **Si usted decide participar, tiene opción de cancelar en cualquier momento sin afectar esa relación.**

Aproximadamente 20 sujetos pueden estar involucrados en esta investigación en UIC.

¿Cuál es el objetivo de esta investigación?

Investigadores estan buscando de entender más sobre los peligros y riesgos en la salud de mujeres que tejen en su hogar junto con las tareas cotidianas de casa. Investigadores también están interesados en aprendiendo cómo reducir el dolor musculoesquelético en su trabajo de tejer.

¿Qué procedimientos están involucrados?

Esta investigación se llevará a cabo en su casa. Nos gustaría observarla haciendo tareas de tejer en su casa dos veces y luego hacerles algunas preguntas. Cada visita durara dos a tres horas.

Los procedimientos del estudio son:

- Dos visitas de sitio para observar la área en donde hace su trabajo de tejer. Cada visita durara como dos horas. Durante este tiempo, vamos a tomar fotografías y video de sus tareas de tejer y área de trabajo. Las grabaciones de vídeo no capturaran sus características faciales. Puede elegir no tener fotografías y/o video tomado y participar en este estudio.
- Una entrevista en profundidad con usted acerca de sus responsabilidades en el trabajo de tejer , las responsabilidades del hogar y sus percepciones de la salud. Esta entrevista tomará alrededor de dos horas y estará audio grabado.

¿Cuáles son los riesgos y molestias potenciales?

De nuestro conocimiento, las cosas que va a hacer no tienen más riesgo de daño de lo que experiencia en la vida diaria. Si algunas preguntas o medidas le hacen sentir incómodas, puede elegir no contestarlas. Si las fotografías o grabaciones de vídeo las hace sentir incómoda, puede elegir que no se toman. Usted puede retirarse en cualquier momento.

Se me informará acerca de nueva información que pueda afectar mi decisión de participar?

Durante el transcurso del estudio, se le informará de cualquier resultado significativo nuevo de investigación (ya sea bueno o malo), tales como los cambios en los riesgos o beneficios derivados de la participación en la investigación o nuevas alternativas a la participación, que podrían causar que usted cambie su decisión acerca de continuar en el estudio. Si recibe información nueva, deberá obtenerse de nuevo su consentimiento para seguir participando en este estudio.

¿Hay beneficios de tomar parte en la investigación?

Es posible que participación en este estudio de investigación no le beneficiará personalmente, pero nosotros [los investigadores] podemos aprender cosas nuevas que ayudarán a los demás.

¿Qué otras opciones existen?

Usted tiene la opción de no participar en este estudio

¿Qué pasa con la privacidad y la confidencialidad?

Las personas quienes saben que usted es sujeto de investigación son miembros del equipo de investigación. Además, personal de VOZ y cualquier persona en su casa durante las visitas de su espacio de trabajo pueden saber que esta participando en un proyecto de investigación. Así que ninguna información acerca de usted será compartida con otros sin su autorización por escrito, o si necesario, para proteger sus derechos o el bienestar (por ejemplo, cuando la Oficina UIC para la Protección de Sujetos de Investigación supervisa el proceso de investigación o consentimiento) o si es requerido por la ley. Se le asignará un identificador único y su grabación de audio será trasladado a un programa de software en el ordenador del PI .

Este estudio consiste en la grabación de audio de su entrevista con la investigadora. Ni su nombre ni ninguna otra información de identificación se asociarán con las grabaciones de audio o la transcripción. Sólo el equipo de investigación podrá escuchar las grabaciones. Usted tendrá la oportunidad de revisar las grabaciones de audio y transcripciones.

El equipo de investigación transcribirá la grabación. Una transcripción de la entrevista estará disponible 10 días después de su entrevista. Usted tendrá 14 días para revisar su transcripción. Si decide revisar la transcripción, usted tendrá la oportunidad de aclarar la información que usted ha proporcionado, solicitar que ciertas citas no se pueden utilizar, y retirar el consentimiento por completo del proyecto. Las transcripciones y grabaciones de audio serán destruidos tras la finalización del estudio.

Información del estudio que le identificara individualmente y el formulario de consentimiento firmado por usted serán examinados o copiados para analizar la investigación por el Instituto Nacional para la Seguridad y Salud Ocupacional (NIOSH)

Un posible riesgo de la investigación es que su participación en la investigación o información sobre usted y su salud podría ser conocida a personas ajenas a la investigación. Se le asignará un número de identificación único y la información que vincula al identificador único se mantendrá en un archivo protegido por contraseña separada. Sólo el equipo de investigación tendrá acceso a esta información . Al finalizar el proyecto, se destruirán todos los datos.

Cuando los resultados de la investigación se publican o discutidos en conferencias, ninguna información se incluirá que revelaría su identidad. El equipo de investigación tendrá acceso a sus entrevistas de audio. Usted tiene el derecho de revisar las cintas de audio de su entrevista y estas cintas serán destruidos una vez completado el estudio.

¿Cuáles son los costos para participar en esta investigación?

No hay costos para usted por participar en esta investigación.

¿Me reembolsarán por cualquiera de mis gastos o me pagarán por mi participación en esta investigación?

Recibirá U$10,00 (7.085 pesos chilenos) para cada visita de estudio completado. Si no termina el estudio, será compensado solo por las visitas que ha completado. Si completa el estudio, recibirá un total de U$20,00 (14.170 pesos chilenos). Recibirá su pago dentro de inmediatamente después de completar las visitas y la entrevista.

¿Puedo retirarme o ser eliminada del estudio?

Si usted decide participar, es libre de retirar su consentimiento y discontinuar participación en cualquier momento.

Elegir no participar en este estudio o de dejar de ser en este estudio no resultara en ninguna sanción a usted o pérdida de beneficios a los que tiene derecho. Específicamente su decisión de no estar en este estudio no afectará la relación presente o futuro con VOZ.

Los investigadores también tienen el derecho de cancelar su participación en este estudio sin su consentimiento si ellos creen que es en su mejor interés.

En el caso de que usted se retire o se le pide dejar el estudio, usted todavía será compensado como se describió anteriormente.

¿A quién debo contactar si tengo preguntas?

Póngase en contacto con los investigadores Gabriela Gracia, MSc. a ggraci2@uic.edu o Dr. Hugo Romero a +56 45 22054 58 o hugo.romero@uct.cl o Jeanette Pérez Comité Ética de la Investigación de la Universidad Católica de Temuco jeperez@uct.cl:

- Si tiene alguna pregunta acerca de este estudio o su participación en él
- Si tiene preguntas, inquietudes o quejas sobre la investigación.

¿Cuáles son mis derechos como sujeto de investigación?

Si usted siente que no ha sido tratado de acuerdo con las descripciones en este formulario, o si tiene alguna pregunta sobre sus derechos como sujeto de investigación, incluyendo preguntas, preocupaciones, quejas, o para ofrecer de entrada, puede llamar a la Oficina para la Protección los Sujetos de Investigación (OPRS) por correo electrónico al uicirb@uic.edu o contactar Dr. Hugo Romero de la Universidad Católica de a +56 45 22054 58 o hugo.romero@uct.cl o Jeanette Pérez Comité Ética de la Investigación de la Universidad Católica de Temuco jeperez@uct.cl.

Recuerde:

Su participación en esta investigación es voluntaria. Su decisión sobre su participación no afectará sus relaciones actuales o futuras con la Universidad. Si decide participar, usted es libre de retirarse en cualquier momento sin afectar dicha relación.

Comprensión de la investigación:

Voy a hacerle algunas preguntas sobre lo que acaba de leer o lo que acabamos de revisar

- Nombre al menos dos cosas que se espera de usted durante el estudio.
- Explique lo que va a hacer si usted experimenta malestar o incomodidad durante el estudio.
- ¿Tiene que participar en este estudio o puede decir "no"?
- ¿Cuál es el propósito de este estudio?

Firma del Sujeto

He leído (o alguien me ha leído) la información anterior. Se me ha dado la oportunidad de hacer preguntas y mis preguntas han sido contestadas a mi satisfacción. Estoy de acuerdo en participar en esta investigación. Se me dará una copia de este formulario firmado y fechado.

Sus iniciales ___________ indican su permiso para ser fotografiado

Sus iniciales ___________ indican su permiso para ser video grabado.

________________________________ ______________

Firma Fecha

____

Nombre—en Letra Impresa

____ ____________________

Firma de la persona que obtiene el consentimiento Fecha (debe ser igual que del sujeto)

____

Nombre de la persona que obtiene el consentimiento—en Letra Impresa
